# *ZNF827* pleiotropic cardiovascular risk locus involves regulation by nuclear factor-1

**DOI:** 10.1042/CS20257956

**Published:** 2026-05-05

**Authors:** Yingwei Liu, Lu Liu, Asraa Esmael, Alberto Tezza, Charlie London, Margaux-Alison Fustier, Adrien Georges, Nabila Bouatia-Naji

**Affiliations:** 1 Université Paris Cité, Inserm, PARCC, F-75015 Paris, France; 2 Stanford Cardiovascular Institute, Stanford University School of Medicine, CA, U.S.A.; 3 Division of Vascular Surgery, Department of Surgery, Stanford University School of Medicine, CA, U.S.A.

**Keywords:** Functional genomics, genetic risk cardiovascular disease, spontaneous coronary artery dissection, transcriptional regulation

## Abstract

Spontaneous coronary artery dissection (SCAD) is a form of myocardial infarction that predominantly affects middle-aged women, caused by the spontaneous onset of an intramural hematoma leading to heart ischemia. SCAD genetic risk loci, such as the* ZNF827* locus on chromosome 4, were previously associated with the risk for coronary artery disease, systolic blood pressure, and ascending aortic diameter variability, but the molecular processes driving these genetic associations are unknown. In the present study, we demonstrated that these genetic associations were colocalized and could all be explained by the intronic common variant rs13128814, which overlapped epigenetic regulatory markers specifically active in vascular smooth muscle cells (SMCs) and fibroblasts. Using reporter assay experiments, we found that the SCAD-risk allele (rs13128814-A) was associated with increased transcriptional activity in A7r5 SMCs. *In silico* predictions and reporter assays suggested nuclear factor-1 (NF1) transcription factors to preferentially bind to SCAD risk allele. We found that SCAD genetic association colocalized with a *ZNF827* eQTL association in artery tissues. Knockdown of *ZNF827* in human iPSC-derived SMCs and fibroblasts identified a large number of dysregulated genes enriched in relevant pathways such as macroautophagy and insulin signaling. Our findings support the NF1-dependent rs13128814 effect on the expression of *ZNF827* as a potential molecular mechanism underpinning multiple cardiovascular trait genetic risk loci. *ZNF827* may act as a broad regulator of gene expression in vascular SMCs and fibroblasts. Further investigation using multiple cell types, organoids, and *in vivo* models may clarify the implications of *ZNF827* in arterial fragility observed in SCAD and other arterial diseases.

## Introduction

Spontaneous coronary artery dissection (SCAD) is an acute coronary syndrome primarily affecting young women, representing up to 35% of acute coronary syndromes in women under the age of 50 [1,2]. Unlike atherosclerosis-related coronary artery disease (CAD), SCAD results from a hematoma obstructing the coronary artery at the origin of the arterial dissection [1]. The pathophysiological mechanisms of SCAD remain unclear. While traditional cardiovascular risk factors like aging, smoking, high blood pressure, and high cholesterol levels are associated with myocardial infarction involving atherosclerotic CAD [3,4], SCAD primarily affects younger, often healthy individuals, which challenges the usefulness of these risk factors to identify patients at higher risk [5,6]. This has motivated the investigation of genetic predisposition factors to better understand the etiology of SCAD.

We have recently performed a large meta-analysis of genome-wide association studies (GWAS) for SCAD, where we reported 16 genetic risk loci [7]. Multiple genes involved in extracellular matrix synthesis and maintenance, smooth muscle cell (SMC) contraction, and tissue-mediated coagulation were highlighted as potential target genes in SCAD risk loci [7]. All identified variants were located in non-coding genomic regions (intronic or intergenic), with causal variants and target genes remaining to be unambiguously identified at most SCAD loci*.*

Interestingly, we showed that the genetic basis of SCAD is shared with several and sometimes more common cardiovascular diseases and traits. For example, three of SCAD loci are consistently associated with fibromuscular dysplasia, a non-atherosclerotic systemic arteriopathy that occurs in at least 50% of SCAD patients [2,8], and migraine, commonly reported by SCAD patients [9]. SCAD loci are also frequently associated with CAD, stroke, and dysregulated blood pressure.

Among newly identified SCAD genetic risk loci, the *zing finger protein 827* (*ZNF827*) locus stands out as particularly paradigmatic, with several GWAS signals previously reported at the locus. The strongest association signal with SCAD is located within the 4th intron of *ZNF827*, which encodes a zinc finger protein predicted to bind nucleic acids. Previous studies pointed to potential functions of *ZNF827* acting as a major regulator both through its RNA and protein products [10–12]. However, the regulatory mechanisms at the associated non-coding variants and the putative role of *ZNF827* as a target gene in this locus remain unexplored.

In the present study, we aimed at clarifying the potential mechanisms linking genetic variants at *ZNF827* locus with the risk for several cardiovascular diseases. First, we identified one intronic variant as the most likely causal variant shared between SCAD and several other cardiovascular diseases and traits through multi-trait colocalization analyses. Second, we assessed the potential transcriptional regulatory features of this variant using reporter assays. Third, we aimed to unambiguously determine the target gene(s) at *ZNF827* locus. Finally, we examined the transcriptional effect of *ZNF827* knockdown in vascular SMCs and fibroblasts. Altogether, our results provide an exploration of regulatory mechanisms at a pleiotropic genetic locus involved in vascular diseases.

## Methods

### Genetic lookup and colocalization

Proxies of SCAD lead SNP rs1507928 were retrieved from the 1000 Genomes reference panel using the LDProxy function of LDLinkR package (v1.3.0), using European populations and GRCh37 genome build. SNPs with *r*^2^ >0.7 were used to probe the GWAS Catalog database (downloaded on 2023 October 11). Traits with genome-wide association (*P_leadSNP_* <5×10^−8^) and direct cardiovascular relevance were retained for further analyses. Summary statistics were retrieved from individual studies [7,13–16]. Multitrait colocalization was performed using HyPrColoc package (v1.0) [17] with default options. All traits clustered together, and the global posterior probability for these traits to share a single causal variant was reported. SNP score (posterior probability of being causal) was also used to identify potential causal SNPs. For colocalization of SCAD association with *ZNF827* eQTL, signal colocalization was evaluated using R coloc package (v5.1.0) with default values as priors [18]. eQTL associations for all variants were retrieved from GTEx website (v10, gtexportal.org). The H4 coefficient indicating the probability of the two traits sharing a causal variant was reported. Colocalization plot was generated using locuscomparer package (v1.0.0).

### Epigenomic data

Single-nucleus ATAC-Seq in coronary arteries was retrieved from the Sequence Read Archive (GSE175621) [19]. Clustering and cell type attribution were performed following the pipeline provided by the authors using ArchR package (v1.0.1). Fragments attributed to each cell type were retrieved in bed format, converted to bam format using bedtools BedtoBam (v2.3.0), and bigwig coverage files were generated using deeptools bamcoverage (v3.5.4). Coronary Artery RNA-Seq dataset (raw counts) was retrieved from the GTEx website. Read normalization was performed using DESeq2 package (v1.38.3). Coronary Artery Histone ChIP was retrieved from ENCODE (H3K4me3: ENCFF811RQX, H3K27Ac: ENCFF130NUG). Coronary Artery ATAC-Seq, Human Dermal Fibroblast, and Coronary Artery SMC were retrieved from the Sequence Read Archive (Bioprojects PRJNA295524 and PRJNA69000) and analyzed as previously described [20]. RNA-Seq and ATAC-Seq datasets during iPSC differentiation into SMC were retrieved from the Sequence Read Archive (Bioproject PRJNA899672) and analyzed as previously described [21]. We used Integrated Genome Browser (IGB, v10.1.0) to visualize read density profiles and peak positions in the context of human genome [22].

### Cell culture

Rat smooth muscle cells (A7r5) were acquired from ATCC (Manassas, Virginia), and grown in DMEM with the addition of 10% FBS from Thermo Fisher Scientific in Waltham, Massachusetts, U.S.A. BJ fibroblasts were acquired from ATCC (Manassas, Virginia), and grown in DMEM with the addition of 10% FBS from Thermo Fisher Scientific (Waltham, Massachusetts, U.S.A.). Human iPSC line SKiPS-31.3 was obtained by reprogramming of human dermal fibroblast of a healthy male adult volunteer as previously described [23]. iPSC lines 11.10 and 12.10 were purchased from Cell Applications (San Diego, CA). All iPSC lines were nurtured in mTeSR Plus medium (STEMCELL Technologies). The iPSCs were differentiated into mesoderm-derived vascular SMCs over a 24 days differentiation protocol as previously described [24]. Briefly, iPSCs (Day 0) were dissociated into single cells, seeded on Matrigel-coated dish cultured in E8BAC medium (E5 medium plus 5 ng/ml BMP4, 25 ng/ml Activin A, 19.4 mg/l insulin, 10 μM Y27632 and 1 μM CHIR99021) for 36 hours. Cells were dissociated again, seeded at low density on Matrigel and in grown in E6T medium (E5 medium supplemented with 19.4 mg/l insulin, 1.7 ng/ml TGF-β1 and 10 μM Y27632) for 18 hours (Day 3). Medium was then replaced with E5F medium (E5 medium supplemented with 19.4 mg/l insulin and 100 ng/ml FGF2) until day 8. From day 8 to day 11, the cells were treated with FVR medium (E5 medium supplemented with 19.4 mg/l insulin, 50 ng/ml VEGF and 5 μM RESV). Cells were then treated with E6-R medium (E5 medium supplemented with 19.4 mg/l insulin, 5 μM RESV, 25 μM Repsox) for 12 days, with a dissociation on day 16. After reaching the 24-day mark, iPSC-derived SMCs were maintained in E6-R medium, all of which were sourced from Thermo Fisher Scientific (Waltham, MA, U.S.A.). Expression of SMC markers was verified using qPCR.

### Data visualization and statistical analyses

Unless otherwise noted, all statistical analyses and figures were performed in R (v4.2.2), using following packages: ggplot2 (v3.4.4), rtracklayer (v1.58.0), ggrepel (v0.9.3), data.table (v1.14.8), dplyr (v1.1.3), tidyr (v1.3.0). For transcription factor binding site (TFBS) prediction, a 51 bp sequence centered on rs13128814 was used as input to PERFECTOS-APE webserver (opera.autosome.org/perfectosape/scan), using HOCOMOCO-*in vivo* (v12) collection of position weight matrices as reference database [25].

### Dual-luciferase enhancer reporter assay

To assess promoter activity, dual-luciferase reporter assays were conducted. Using BglII/HindIII restriction enzymes, the TK-minimal promoter was removed from pRL-TK (Promega, Madison, Wisconsin, U.S.A.) and inserted into the appropriate locations of pGL4.12 (Promega, Madison, Wisconsin, U.S.A.) to create the pGL-TK minimal promoter reporter plasmid. Between the Nhe1 and Xho1 sites of pGL-TK, a 947 bp DNA fragment including rs13128814 was amplified from the mix of human genomic DNA (primers: F: gctcgctagcACCTTTAAGTCTCGGCCTCC, R: tatcctcgagTCCCGGGTTCAAGCTATTCT). To generate rs13128814-A pGL4-TK plasmids, fusion PCR was performed with primers covering the variant (F: GGGCAGAGAACAGAGCCAAGTTCCTGTTTTGCTGC, R: GCAGCAAAACAGGAACTTGGCTCTGTTCTCTGCCC). A7r5 were seeded in 96-well plates with a concentration of 10,000 cells per well and, after 24 h, transfected with luciferase reporter constructs or containing the relevant promoter regions using FuGENE HD Transfection Reagent Complex (Promega, Madison, Wisconsin, U.S.A.). pCMV-NFIA1.1 (a kind gift from Richard Gronostajski, Addgene plasmid #112698 [26]) was transfected with luciferase-expressing plasmids at the same time for the NFIA overexpression condition. Forty-eight hours after transfection, the Firefly and Renilla luciferases were measured by performing the Dual-Luciferase Reporter Assay System (Promega, Madison, Wisconsin, U.S.A.), following the instructions of the manufacturer. Signal was recorded using the Mithras LB 940 Multimode Microplate Reader machine (Berthold Technologies, Bad Wildbad, DE). The enhancer capacity was calculated by the ratio of Firefly to Renilla luciferase. Statistical significance was evaluated using an R studio *t*-test with unequal variances.

### siRNA knockdown

In six-well plates, iPSC-derived SMCs (SKiPS-31.3 and 12.10 lines) and BJ fibroblast cells were seeded at a density of 100,000 cells per well to reach a roughly 70%–80% confluence for the siRNA knockdown tests. Silencer® Select N°1 negative control siRNA or predesigned Silencer® Select siRNAs for ZNF827 (s45694, s45695, and s45696), NFIA (s9476), NFIB (s9494), NFIC (s9497), and NFIX(s9501) (Thermo Fisher Scientific, Waltham, MA, U.S.A.) were used for the transfection process. For the 72-h transfection process, Opti-MEM and Lipofectamine RNAiMAX transfection reagent (Thermo Fisher Scientific, Waltham, MA, U.S.A.) were used.

### Quantitative RT-PCR analysis

Cells were collected and given a PBS rinse before being subjected to the quantitative RT-PCR analysis. RNA purification was carried out following the manufacturer’s instructions, utilizing the RNeasy Plus Mini kit (Qiagen, Hilden, Germany). Reverse transcription was carried out using the iScript cDNA Synthesis Kit (Bio-Rad, Hercules, California, U.S.A.). The final cDNA was used as a template for quantitative real-time PCR with the GoTaq qPCR master mix (SYBR Green), purchased from Promega (Madison, WI, U.S.A.). The PCR reactions were carried out on an Applied Biosystems StepOne Plus device, and primers were designed to detect the *ZNF827* mRNA level produced using the internet tool https://primer3.ut.ee/ (F: TTTGAGTGTGATGTGTGCCA, R: ACCACTGTCCTGAGTTTCCT). The comparative CT approach in StepOne software was utilized to ascertain the relative amounts of gene expression. The housekeeping genes *GAPDH*, *ACTB*, and *SDHA* expression levels were used to standardize the results. To ensure accuracy, each reaction was carried out in triplicate. A two-sample *t*-test with unequal variances was used to determine the statistical significance of each result.

### RNA-Seq

PolyA+ mRNA libraries were prepared from 100 ng to 1 μg of total RNA using the QIAseq Stranded RNA library preparation kit (Qiagen) according to the manufacturer’s instruction. Libraries were sequenced on a NextSeq500 instrument (Illumina, San Diego, CA, U.S.A.) using NextSeq 500/550 High Output Kit v2 (75 cycles). Reads were demultiplexed using bcl2fastq2 (v2.18.12), and adapters were trimmed using Cutadapt (v1.15). Reads were then mapped to human genome (GRCh38.104) using the STAR aligner (v2.7.9a) with the following options: --outFilterType BySJout --outFilterMultimapNmax 20 --alignSJoverhangMin 8 --alignSJDBoverhangMin 1 --outFilterMismatchNmax 999 --outFilterMismatchNoverReadLmax 0.04 --alignIntronMin 20 --alignIntronMax 1000000 --alignMatesGapMax 1000000. We used per gene read counts as direct input for differential expression analysis using DESeq2 (v1.38.3) package in R (v4.2.2), keeping only genes with mean read counts over 1, using cell type (SMC or fibroblasts) as a covariate [27]. We transformed the count matrix using variance stabilizing transformation with option blind=FALSE. Differentially expressed genes were determined using res function, and log_2_ fold changes were determined using lfcShrink function with ashr shrinkage estimator [28]. Gene ontology term enrichments were calculated using clusterProfiler package (v4.6.2).

### Cell viability assay

Cells (5000 cells/well) were seeded in 96-immuno white immune plates (Thermo Fisher Scientific, Waltham, MA, U.S.A.) with 16 replicates. CellTiter-Glo 2.0 assay (Promega, Madison, Wisconsin, U.S.A.) was used to measure cell viability. Plates were loaded with CellTiter-Glo reagents, and the luminescent signal was recorded using the Mithras LB 940 Multimode Microplate Reader machine. Measurements of luciferase were performed 4, 24, 48, 72, and 144 h after transfection. The viability of all luciferase measurements was normalized to the values at 4 h. Statistical significance was evaluated using the Wilcoxon rank sum test.

### Cell migration assay

One hundred microliters of cell suspensions (100,000 cells/ml) were plated on the top of the filter membrane in the Transwell insert (24-well Transwell chamber with 8 μm pore size, Corning, New York, U.S.A.). Six hundred microliters of culture medium (normal or supplemented with 0.2 μg/ml recombinant human PDGF-BB; Thermo Fisher Scientific, Waltham, MA, U.S.A.) were added into the bottom chamber. Chambers were incubated under the cell culture condition for 20 h. The inserts were then collected, fixed in 70% ethanol, and dyed with 0.2% crystal violet for 15 min. The migrated cells were imaged and counted using the ImageJ tool. Statistical significance was assessed using the Wilcoxon rank sum test.

### Western blotting

After cells were washed once by PBS, 100 μl of 2× Laemmli Sample Buffer (Bio-Rad, Hercules, California, U.S.A.) was added for 12-well plates. Samples were collected and incubated at 95°C for 10 min, supplemented with 5% β-mercaptoethanol. The protein samples were separated on 4%–20% mini-Protean TGX gels (BIO-RAD, Hercules, California, U.S.A.) and transferred to nitrocellulose membranes. Membranes were blocked with TBST buffer supplemented with 5% (w/v) milk and incubated at 4°C overnight with primary antibodies (1:1000 in 2% BSA buffer): mouse anti-smooth muscle α-Actin (Santa Cruz Biotechnology, Dallas, Texas, U.S.A.), rabbit anti-TAGLN (Abcam, Cambridge, U.K.), rabbit anti-MYH11 (Abcam, Cambridge, U.K.), and rabbit anti-Calponin 1 (Abcam, Cambridge, U.K.). After incubating with HRP-conjugated secondary antibodies (BIO-RAD, Hercules, California, U.S.A.), membranes were revealed with SuperSignal West Pico PLUS chemiluminescent substrate (Thermo Fisher Scientific, Waltham, MA, U.S.A.) and visualized by the FujiFilm LAS-4000 mini system (FUJIFILM, Minato City, Tokyo, Japan).

## Results

### A single variant may cause an association with multiple cardiovascular traits and diseases at *ZNF827* locus

**Figure 1:**
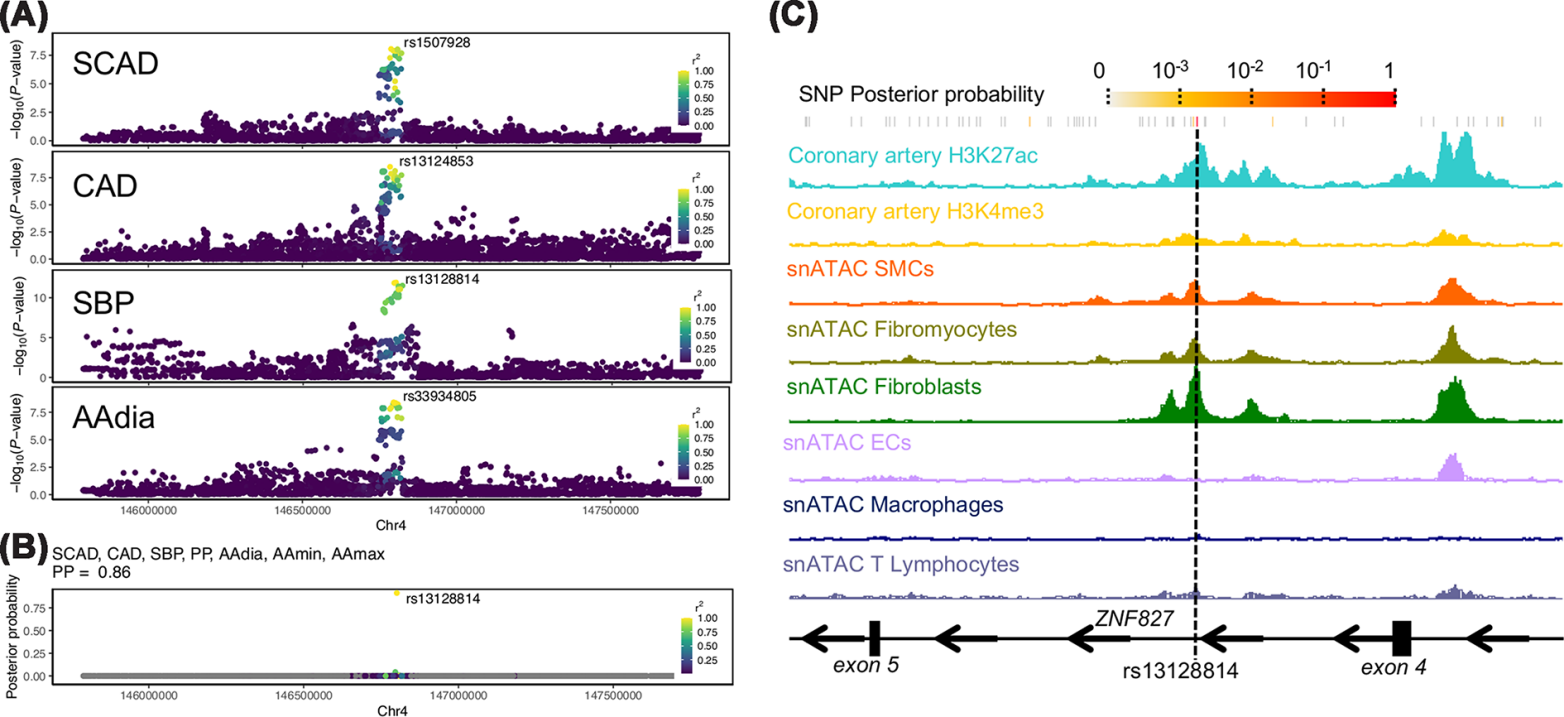
Genetic associations and functional annotation at ZNF827 locus (**A**) Local genomic plots represented the genetic association of common variants in *ZNF827* locus to SCAD, CAD, SBP, and AAdia. Top variants are indicated for each association signal, and blue to green indicates linkage disequilibrium increasing levels (*r*^2^ in the European population of the 1000 Genomes reference panel) of variants to the top one. (**B**) Bayesian posterior probability for each variant in *ZNF827* locus as causal for the associations with SCAD, CAD, SBP, PP, AAdia, and ascending aorta minimal and maximal areas (AAmin, AAmax). (**C**) Genome browser visualization of histone chromatin immunoprecipitation and single nuclei snATAC-Seq read densities in the region surrounding rs13128814. The dashed grey line highlights rs13128814’s exact position.

In our previous study, the *ZNF827 locus* was found to be associated with SCAD, with rs1507928 as the lead variant. To identify whether other traits or diseases could potentially be associated with the same genetic variant, we looked up the GWAS catalog for associations involving variants in high linkage disequilibrium (LD) with SCAD lead variant rs1507928 (*r*^2^ >0.7 in the European population of the 1000G reference panel, Supplementary Table S1). We found seven cardiovascular traits and diseases, including CAD [29]; systolic blood pressure (SBP) [30]; pulse pressure (PP) [30]; and ascending aorta diameter (AAdia), maximum area (AAmax), and minimum area (AAmin) [15] (Table 1). To determine whether these different associations may be caused by a unique and the same variant, we retrieved summary statistics of all seven cardiovascular traits or diseases. All associations involved multiple non-coding genetic variants in high LD between each other (Figure 1A and Supplementary Figure S1). Using HyPrColoc multi-trait colocalization, we found that all seven traits cluster together, with an overall probability to share a single causal variant estimated at 85% (Figure 1B). Under this hypothesis, a single variant (rs13128814) was defined as the most likely causal variant for all seven association signals (PP_rs13128814_ = 0.91; Figure 1B). SCAD risk allele (rs13128814-A) was associated with reduced CAD risk, decreased SBP and PP, and increased ascending aorta measurements (Table 2). Rs13128814 is located within the 4th intron of *ZNF827*, in a genomic region that we found to overlap with H3K4me3 and H3K27ac histone marks in human coronary artery ChIP-seq datasets from the ENCODE consortium (Figure 1D). Using single-nucleus chromatin accessibility maps from diseased and healthy coronaries [19] (Supplementary Figure S2), we found that this regulatory element overlapped with open chromatin regions in coronary artery SMCs, fibromyocytes, and fibroblasts. However, this regulatory element was not accessible in coronary endothelial and immune cells (Figure 1D).

**Table 1:** Lookup for lead SNPs in high LD with SCAD lead SNP in GWASes for cardiovascular traits and diseases

Trait	lead SNP	Chr	Pos	A1	A2	β	SE	P	r2	Correlated Alleles
Spontaneous coronary artery dissection (SCAD) [7]	rs1507928	4	146788035	C	T	0.23	0.039	8.9 × 10^−9^	1.00	T = T C = C
Coronary artery disease (CAD) [13]	rs13124853	4	146784774	G	A	−0.03	0.005	3.1 × 10^−9^	0.97	T = A C = G
Systolic blood pressure (SBP) [14]	rs13128814	4	146801002	A	G	−0.22	0.031	1.2 × 10^−12^	0.71	T = G C = A
Pulse pressure (PP) [14]	rs4835266	4	146821725	C	T	−0.16	0.021	4.2 × 10^−15^	0.87	T = T C = C
Ascending aorta diameter (AAdia) [15]	rs33934805	4	146796087	G	A	0.04	0.007	4.0 × 10^−9^	0.98	T = A C = G
Ascending aorta maximum area (AAmax) [16]	rs1979974	4	146800815	G	A	0.05	0.007	3.7 × 10^−9^	0.97	T = A C = G
Ascending aorta minimum area (AAmin) [16]	rs17020769	4	146800922	T	C	0.04	0.007	6.5 × 10^−10^	0.97	T = C C = T

Table indicates the summary statistics for lead variants of association to seven cardiovascular traits and diseases at *ZNF827* locus (+/- 1 Mb of SCAD lead variant). Chr: Chromosome. Pos: Position (hg19 coordinates). A1: effect allele. A2: risk allele. β: Genetic association regression coefficient. SE: Standard error of β. P: Association *P*-value. *r*^2^: Linkage disequilibrium correlation coefficient of lead SNP to rs1507928 (SCAD lead SNP) in European populations (1000 Genomes reference panel). Alleles were aligned so that effected allele is positively correlated with SCAD risk allele for each variant.

**Table 2:** Summary statistics for predicted common causal variant rs13128814 in seven cardiovascular traits and diseases

Trait	rsID	Chr	Pos	A1	A2	β	SE	P
SCAD	rs13128814	4	146801002	A	G	0.22	0.039	3.2 × 10^−8^
CAD						−0.03	0.005	5.9 × 10^−8^
SBP						−0.22	0.031	1.2 × 10^−12^
PP						−0.16	0.021	4.2 × 10^−14^
AAdia						0.04	0.007	7.0 × 10^−9^
AAmax						0.04	0.007	6.9 × 10^−9^
AAmin						0.05	0.007	6.8 × 10^−10^

Table indicates the summary statistics for rs13128814 variant in the association to seven cardiovascular traits and diseases. Chr: Chromosome. Pos: Position (hg19 coordinates). A1: effect allele. A2: risk allele. β: Genetic association regression coefficient. SE: Standard error of β. P: Association *P*-value.

### rs13128814 risk allele is required for *in vitro* enhancer activity

**Figure 2:**
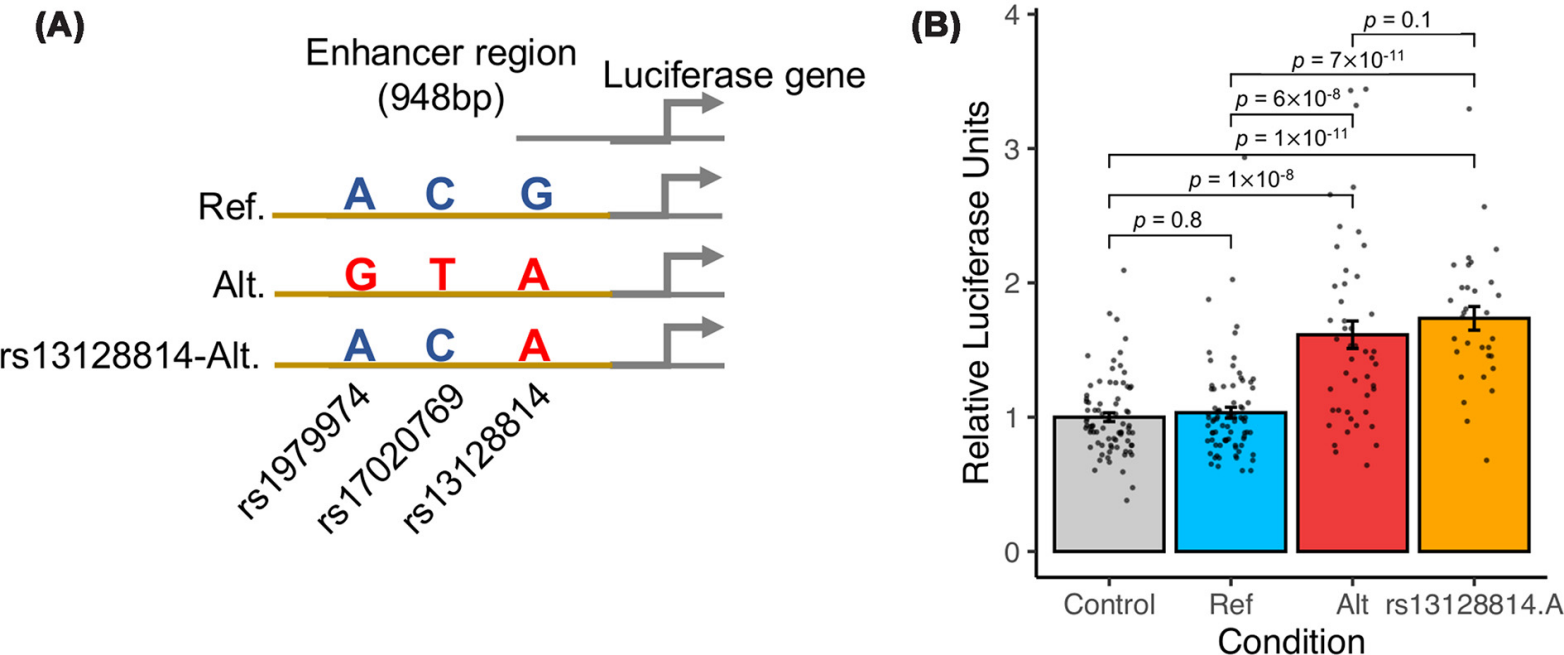
Transcriptional activity of rs13128814-surrounding region (**A**) Illustration of the region encompassing rs13128814 cloned in a luciferase reporter plasmid. rsID and genotype of the three common variants overlapping this DNA fragment are indicated for each construct. (**B**) Luciferase activity measured through overexpression of indicated constructs in A7r5 rat SMCs. *P*-value of the Wilcoxon Rank Sum test (two-tailed) is indicated for each pair of data points.

To determine whether enhancer activity may be affected by rs13128814 genotype, we cloned a 948 bp genomic region centered on the observed open chromatin region in a luciferase reporter plasmid. This region included rs13128814, the most likely causal variant; rs17020769; and rs1979974, two SNPs significantly associated with SCAD at the genomic level and in high LD between each other in European populations (*r*^2^_rs13128814–rs17020769_ = 0.73, *r*^2^_rs13128814–rs1979974_ = 0.73, *r*^2^_rs1979974–rs17020769_ = 1, Figure 2A). First, we compared the transcriptional activity of regions comprising all risk alleles versus the other alleles by transfecting these constructs in A7r5 rat SMCs. The region containing risk alleles was associated with an increased transcriptional activity compared with an empty vector, while the region with the other alleles showed no transcriptional activity (Figure 2B). Then, we compared the activity of the region containing only the variant allele at rs13128814 with the one containing all other alleles. We found an increased transcriptional activity for this region at the same level as all risk alleles (Figure 2B), suggesting that the rs13128814-A allele is driving the transcriptional activity of this regulatory genomic region.

### rs13128814 risk allele creates a binding site NF-1 transcription factors

**Figure 3:**
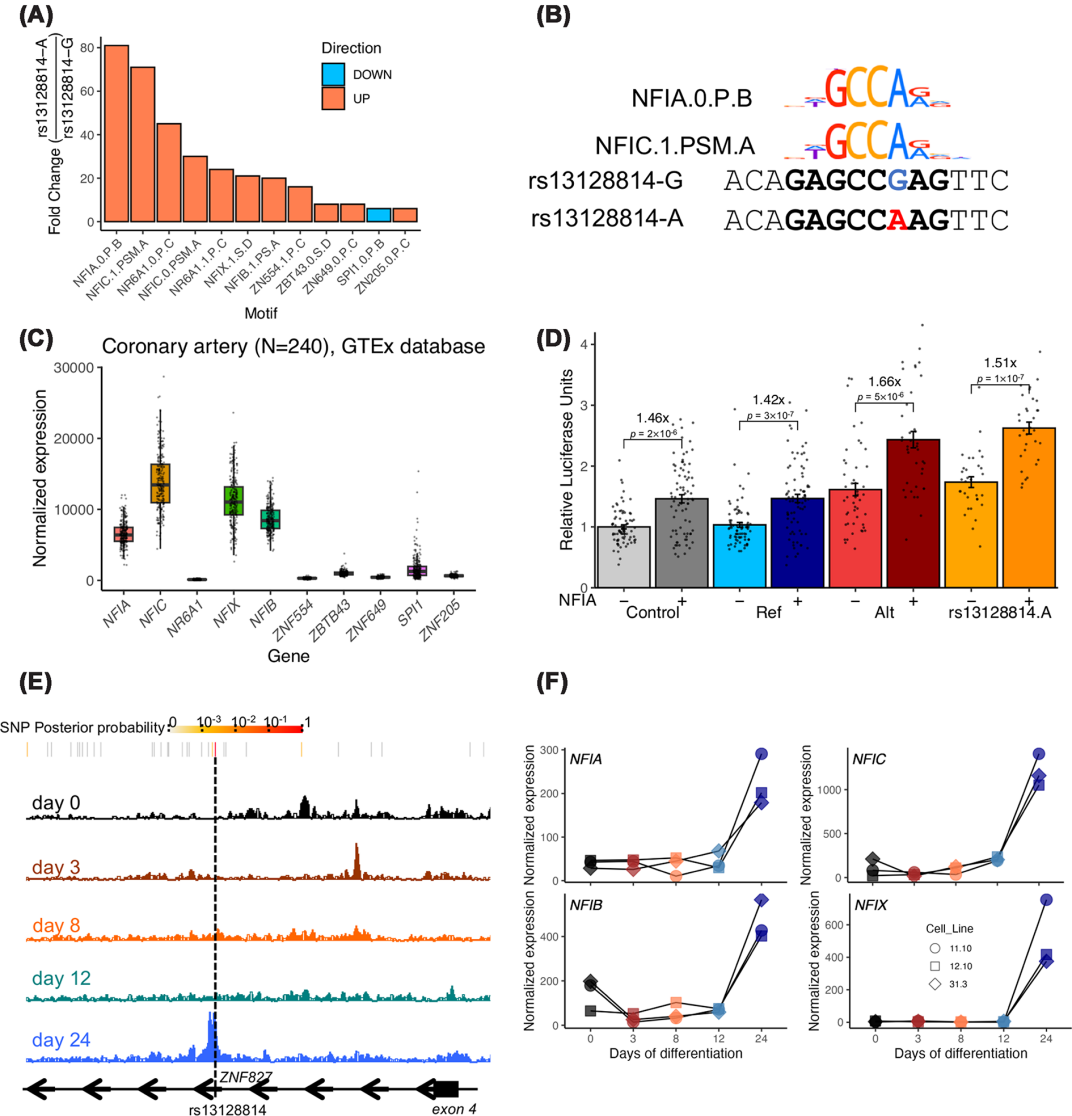
rs13128814 disrupts a potential binding site for NF-1 transcription factors (**A**) Candidate TFBSs affected by rs13128814 allelic variation. Barplot represents the ratio of P-value of similarity of the DNA sequence containing rs13128814-A allele to each TFBS, compared with the alternate allele rs13128814-G. Color indicates if the similarity to TFBS with rs13128814-A is higher (red) or lower (blue). Approximate P-values for position weight matrices were estimated using threshold method as implemented in PERFECTOS-APE package [50]. (**B**) Alignment of DNA sequence close to rs13128814 with NFIA and NFIC binding motifs from HOCOMOCO v12 (‘*in vivo*’ TFBS). (**C**) Normalized expression of candidate transcription factors estimated in 240 coronary artery samples from GTEx database (v10 release). Boxes represent the 25th, 50th, and 75th centiles of the distribution. Lower and upper whiskers indicate a maximal distance of 1.5 times the interquartile range from the first and third quartiles, respectively. (**D**) Luciferase activity measured in A7r5 rat SMCs through overexpression of constructs indicated, with and without pCMV-Nfia. Fold changes are indicated over the graph. *P*-value of the Wilcoxon Rank Sum test (two-tailed) is indicated for NFIA versus control for each reporter construct. (**E**) Genome browser visualization of chromatin accessibility in iPSCs (day 0) and during day 3 to day 24 of SMC differentiation. (**F**) Normalized expression of genes coding for nuclear factor-1 transcription factors in iPSCs (day 0) and during day 3 to day 24 of SMC differentiation.

We used PERFECTOS-APE algorithm to predict TFBS potentially affected by rs13128814 genotype, using *in vivo* sites from HOCOMOCO v12 as candidate TFBS [25]. We found 12 candidate TFBS, corresponding to 10 candidate TFs, predicted to recognize differentially the sequence including rs13128814 (log_2_ Fold Change >4; Figure 3A and Supplementary Table S2). The first two candidate TFBSs corresponded to sites of nuclear factor 1 family (NF-1) NFIA and NFIC, with other differential TFBSs predicted for other NF-1 factors NFIB and NFIX. rs13128814-A allele perfectly matched the NF-1 binding motif, while rs13128814-G disrupted this motif (Figure 3B). We prioritized following up on NF-1 family factors, as they were highly expressed in coronary artery tissues from the GTEx database (v10 release) and particularly present in SMC and fibroblast cell types according to a single-cell RNA-Seq dataset generated from coronary artery tissue [32], while other candidate TFs were poorly expressed in this tissue (Figure 3C), (Supplementary Figure S3). NF-1 factors also appear among key drivers of female-specific regulatory networks in human atherosclerotic plaques of the carotid artery (Supplementary Figure S4), while NF-1 binding sites are part of the top enriched motif in open chromatin of coronary artery SMCs (Supplementary Figure S5). To determine whether NF-1 factors could activate rs13128814-related enhancer, we overexpressed mouse *Nfia* (99.5% identical with human *NFIA*) in the above-described luciferase assay set in A7r5 rat SMCs (Figure 3D). We found that NFIA overexpression increased luciferase expression from the control plasmid, which contained only a minimal promoter region, suggesting NFIA may bind sites in the plasmid backbone (Figure 3D). We, however, observed an increased activation of transcription when NFIA was overexpressed with the plasmid containing rs13128814-A allele but not with the plasmid containing rs13128814-G allele (Figure 3D).

Our recent study supports that the differentiation of vascular SMCs using RepSox, a TGF-β inhibitor, can lead to a regulatory phenotype closer to artery tissue [21]. A look-up in the open chromatin maps generated during iPSC differentiation using RepSox showed that rs13128814-associated regulatory element became accessible during the last step of this differentiation protocol, concomitantly with the increased expression of SMC markers (Figure 3E, Supplementary Figure S6). Interestingly, an increased expression of NF-1 transcription factors was observed as well, further supporting these TFs to be potentially required for the activation of rs13128814-associated regulatory element (Figure 3F). To assess whether NF-1 factors may act on *ZNF827* expression, we knocked down NF-1 factors individually in R-SMCs, and we observed that knockdown of *NFIA*, *NFIB,* and *NFIX* resulted in a moderate increase of *ZNF827* expression. Knockdown of all 4 NF-1 factors resulted in a marked increase of *ZNF827* expression mRNA level, suggesting NF-1 factors are repressors of *ZNF827* in these cells (Supplementary Figure S7).

### *ZNF827* acts as a major regulator of gene expression in SMCs and fibroblasts

**Figure 4:**
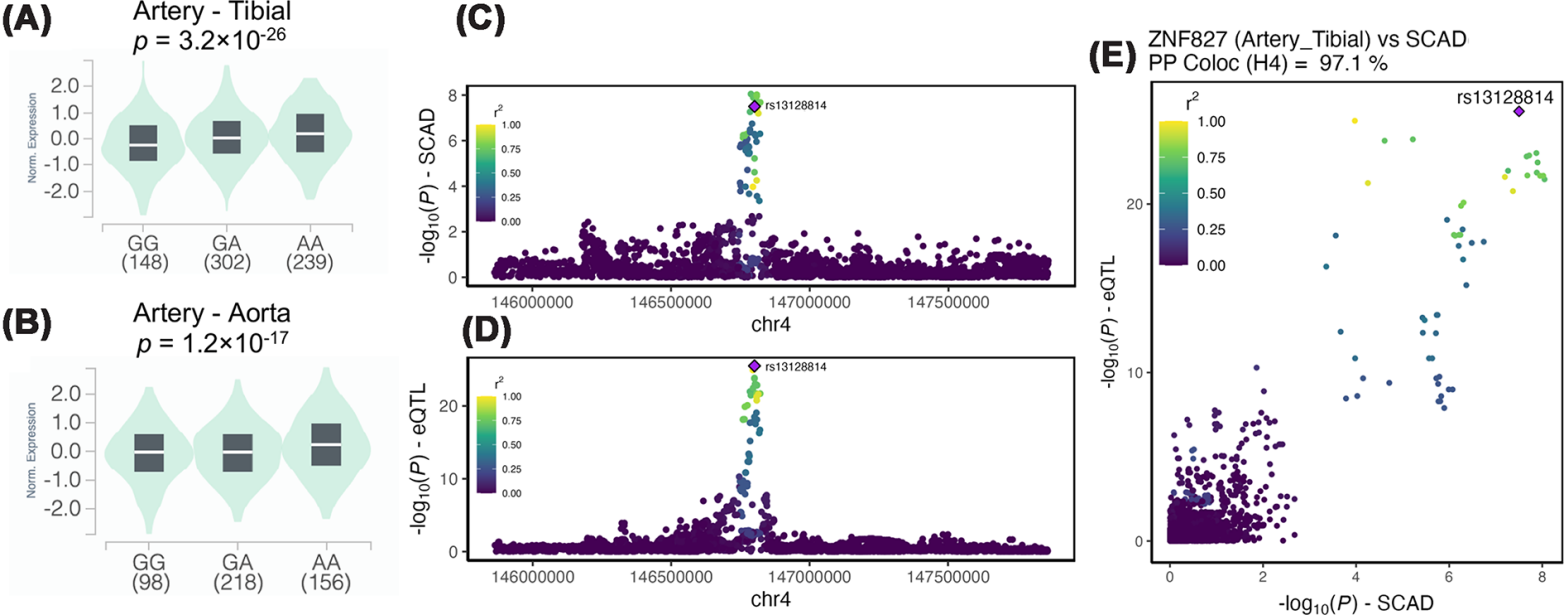
rs13128814 significantly correlated with *ZNF827* expression in artery tissues Violin plots illustrate the association of rs13128814 and *ZNF827* normalized expression in (**A**) tibial artery tissue (*n* = 689, *P* = 3.2 × 10^−26^), and (**B**) aorta (*n* = 472 *P* = 1.2 × 10^−17^). LocusZoom representations of SCAD (**C**) and *ZNF827* tibial artery eQTL (**D**) association at *ZNF827* locus. Dot color represents *r*^2^ of linkage disequilibrium of each SNP in European populations. (**E**) Colocalization plot representing the correlation of SCAD association and ZNF827 tibial artery eQTL association. Dot color represents *r*^2^ of linkage disequilibrium of each SNP in European populations. Probability of the two signals to colocalize on a single causal SNP was estimated to be 97%.

To determine the putative target gene at the *ZNF827* locus, we looked up rs13128814 in the GTEx database (v10 release). We found that rs13128814-A allele was associated with higher expression of *ZNF827* in tibial arteries and aorta, the biggest arterial datasets in GTEx, but not in the coronary arteries dataset (Figure 4A,B). A colocalization analysis at the *ZNF827* locus between genetic association with SCAD and the eQTL data confirmed associations likely to have a single common causal variant in both tibial arteries (PP.H4.abf = 0.97, Figure 4C–E) and aorta (PP.H4.abf = 0.94).

**Figure 5:**
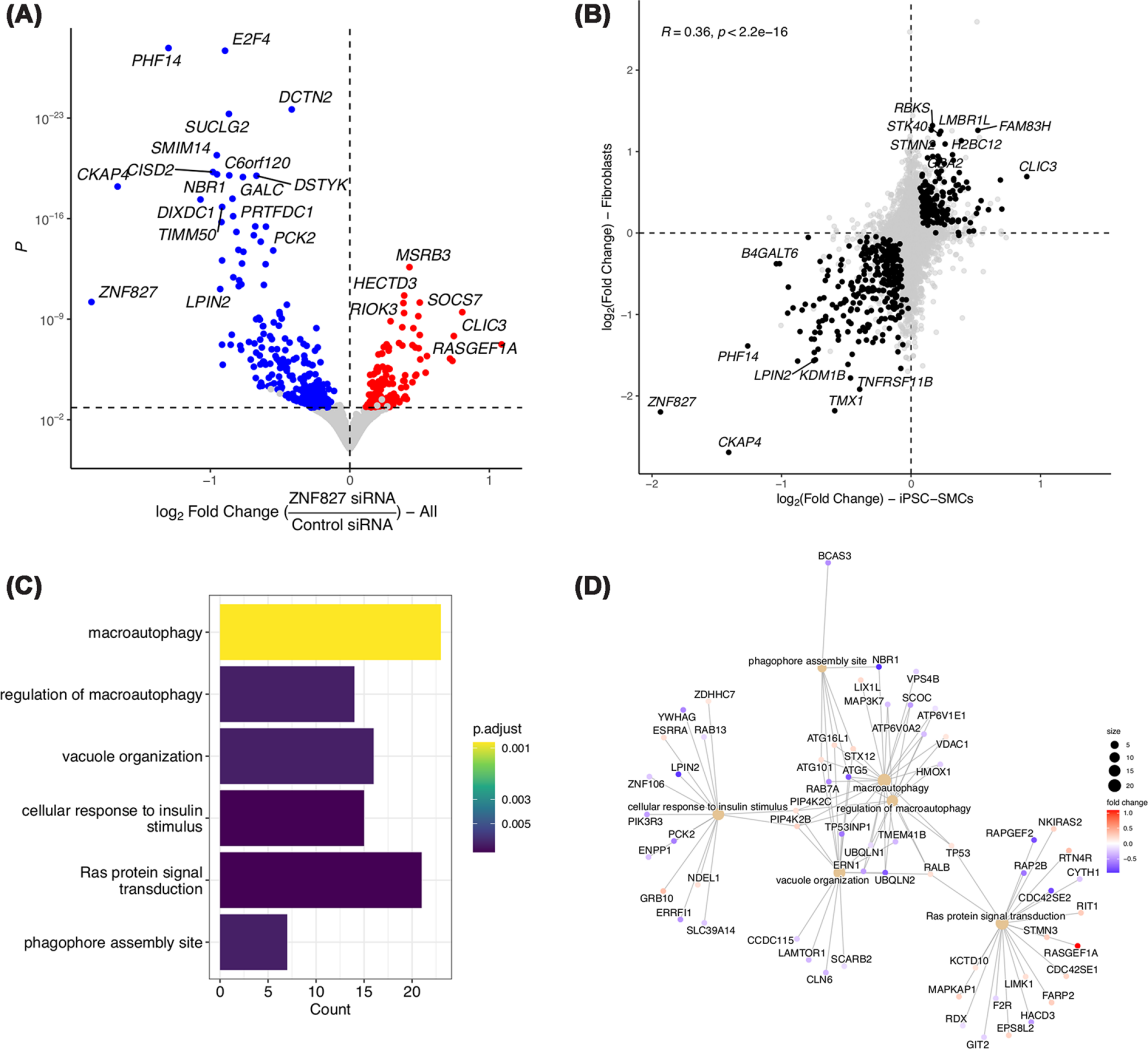
Genes and pathways potentially regulated by ZNF827 in human iPSC-derived SMCs and fibroblasts (**A**) Volcano plot representation of differential expression following ZNF827 knockdown in iPSC-derived SMCs (two clones and three replicates each) and BJ fibroblasts (three replicates). Log_2_ fold changes are represented on the *x*-axis, and *P*-values on log scales are represented on the *y*-axis. Differentially expressed genes (*P.adj* <0.05) are highlighted in blue (down-regulated genes) or red (up-regulated genes). (**B**) Scatterplot representation of differential gene expression in iPSC-derived SMCs (*x*-axis) versus BJ fibroblasts (*y*-axis). Differentially expressed genes consistent between the two cell types are highlighted in black. (**C**) Top enriched gene ontology pathways among differentially expressed genes. Color gradient indicates adjusted P-value of gene set enrichment. (**D**) Network representation of differentially expressed genes involved in enriched pathways. Beige nodes correspond to enriched pathways, with node size correlated to the number of differentially expressed genes. Log_2_ fold-change of differential expression (all samples grouped) is indicated by red-blue color gradient on gene node.

The function of *ZNF827* in arterial tissue is not known. To get insight into its potential role in cardiovascular disease, we knocked down *ZNF827* expression in iPSC-derived SMCs (two clones and three replicates) and BJ primary fibroblasts (three replicates) using siRNAs. We collected RNA 48 h post-transfection, confirmed *ZNF827* knockdown using qPCR (Supplementary Figure S8), and analyzed gene expression using bulk RNA sequencing. The expression of classical SMC markers was not affected by *ZNF827* knockdown in SMCs (Supplementary Figure S9). We identified 437 differentially expressed genes combining data from both SMCs and fibroblasts (*P.adj* <0.05), with most genes (*n* = 270) being down-regulated after the knockdown (Figure 5A, Supplementary Table S3). Differential expression data were significantly correlated between SMCs and fibroblasts (*R* = 0.36, *P* <2 × 10^−16^, Figure 5B). Pathway enrichment analyses showed an overrepresentation of genes involved in the regulation of macro-autophagy, with key autophagy factors like *ATG5* (*Autophagy protein 5*) and *NBR1* (*Next to BRCA1 gene 1*) among the most down-regulated genes (Supplementary Table S4 and Figure 5C,D). Several of the most dysregulated genes following *ZNF827* knockdown are known genes involved in atherosclerosis and arterial disease, such as *caveolin-1* (*CAV1*) [31,32], *osteoprotegerin (TNFRSF11B)* [33,34], *cytoskeleton-associated protein 4 (CKAP4)* [35], and *transcription factor E2F4 (E2F4)* [36].

Despite differential expression being highly correlated between SMCs and fibroblasts, a large group of genes was specifically regulated in fibroblasts (Supplementary Figure S10). Pathway analyses showed an important enrichment of down-regulated genes involved in mitosis and cell cycle regulation, whereas up-regulated genes were enriched in pathways related to protein synthesis (Supplementary Figure S11). Consistently, we observed reduced cellular proliferation in BJ fibroblasts following *ZNF827* knockdown (Supplementary Figure S12). In SMCs, cell viability was not affected immediately following *ZNF827* knockdown, but sustained knockdown led to an increase in cell proliferation (Supplementary Figure S13). *ZNF827* knockdown also decreased SMC ability to migrate in response to PDGF-BB treatment, suggesting that *ZNF827* may participate in tissue remodeling and wound repair.

## Discussion

In the present study, we investigated the regulatory mechanisms and molecular roles of target genes in *ZNF827*, a genetic risk locus previously associated with diverse forms of arterial disease, including ischemic heart disease and traits of blood pressure and aorta size. Our results suggest that all the genetic association signals are likely to be caused by a single common variant located in a regulatory element active in coronary artery SMCs and fibroblasts. *In vitro* experiments support the risk allele of this intronic variant to regulate the expression of an understudied zing finger protein coding gene, *ZNF827*, specifically in arterial wall cells. Findings obtained from iPSC-derived SMCs and primary human fibroblasts support the potential regulatory role of NF-1 family transcription factors. The major transcriptomic changes caused by *ZNF827* knockdown are related to genes involved in atherosclerosis and vascular remodeling, providing plausible clues about the biological mechanisms potentially perturbed in important forms of vascular disease, including SCAD, CAD, and blood pressure.

One key finding in our study is the functional property of the intronic variant in *ZNF827* locus involving transcription factors of the NF-1 family. We found that rs13128814-A allele creates a consensus binding site for members of this transcription factors, whose potential role in vascular biology has not been fully characterized so far. The deletion of NF-1 transcription factors in mice causes perinatal death with a wide range of neurological skeletal developmental default phenotypes but has no cardiovascular consequences [37–40]. Interestingly, NF-1 binding sites are among the most enriched in active regulatory regions of vascular SMCs and endothelial cells [19]. NF-1 factors *NFIA* and *NFIB* were also recently identified as key driver genes in female-specific gene regulatory networks in atherosclerosis, mostly through SMC-related pathways [41]. The four NF-1 genes drive the expression of multiple splice variants incorporating the conserved DNA-binding domain, and the function of most of these variants is unknown [42]. The important regulatory compensation between NF-1 factors, as they recognize the same DNA patterns and show high expression levels in vascular SMCs, may explain the lack of cardiovascular phenotypes following the knockout of individual NF-1 factors [37]. Consistently, we found that knockdown of all four NF-1 transcription factors was required to have a maximum impact on the expression of *ZNF827*. Nevertheless, our findings are consistent with the recent molecular evidence of high enrichment for NF-1 binding sites in genomes of vascular SMCs and motivate deeper investigation to fully understand their potential role in pathological vascular remodeling.

Our *in silico* and *in vitro* studies support *ZNF827*, an understudied zinc finger protein-encoding gene involved in several biological processes, as the most likely target gene at this genetic risk locus for diverse vascular diseases and traits. *ZNF827* is ubiquitously expressed in human tissues and predicted to encode a DNA-binding protein, although its potential function in the vasculature is not known. Previous studies reported ZNF827 as a potential regulator for epithelial-to-mesenchymal transition [10], splicing [10], alternative lengthening of telomeres [43] through homologous recombination [44], and neuronal differentiation [12]. One study showed that the regulatory function of *ZNF827* could be mediated both by its protein product and by a circular RNA, at least in relation to neuronal pathways [12]. Through a cellular knockdown approach, our data support *ZNF827* down-regulation to induce major transcriptional modifications, with a high number of dysregulated genes both in iPSC-derived SMCs (>900) and in fibroblasts (>3000). We hypothesize that the difference in the numbers of dysregulated genes between the two cell types is due to a stronger effect of *ZNF827* knockdown to slow down the cell cycle in fibroblasts, considering that in our iPSC-derived SMC model, cells proliferated at a lower pace [21].

Several genes particularly relevant to vascular disease were among shared regulated genes between iPSC-derived SMCs and fibroblasts. Among these, we cite *TNFRSF11B*, which encodes osteoprotegerin and is a specific marker of fibromyocytes, a subtype of SMCs thought to play a key role in atherosclerosis [33]. Osteoprotegerin was also reported to act as an antagonist of vascular calcification, although its role is complex and involves interaction between vascular SMCs, endothelial cells, and the immune system [34,45]. Another strongly down-regulated gene was *CKAP4*, which encodes the CKAP4, a major player in angiogenesis and SMC function, also known as cytoplasmic protein 63. CKAP4 is a transmembrane and endoplasmic reticulum protein that acts as a receptor for several proteins, including tissue plasminogen activator [46], Wnt inhibitor Dickkopf-1 [47], or nexin 17 [48]. CKAP4 was recently found to regulate vascular calcification and VSMC phenotypic modulation in the context of chronic kidney disease [35]. Among other ZNF827-dependent genes, *CAV1* encodes Caveolin 1, a protein involved in intracellular trafficking that was found to regulate low-density lipoprotein transport and vascular SMCs proliferation in the context of atherosclerosis [31,32]. E2F4 encodes an E2F transcription factor that acts as a repressor of vascular SMC proliferation in intimal hyperplasia [36]. The large number of genes dysregulated and the high relevance of many of them to SMC biology in the context of vascular remodeling designate *ZNF827* as a potential key regulator to prioritize for further investigation involving more complex *in vivo* models.

Our study presents several limitations. First, we did not analyze in detail the binding of NF-1 transcription factor to *ZNF827* intronic region in R-SMCs, the only *in vitro* cell type where we found the regions to be active, due to poor cell proliferation and technical limitations. The effect of NF-1 on *ZNF827* expression is likely context-dependent, as we found it as an activator in rat A7r5 cells, in consistence with eQTL data, while NF-1 knockdown led to increased *ZNF827* expression in iPSC-SMCs. Further analysis will be required to fully elucidate the mechanisms by which NF-1 impacts *ZNF827* expression. Second, we could not investigate the long-term effects of *ZNF827* depletion or overexpression. Our numerous attempts to create knockout cell lines in iPSCs using CRISPR-Cas9 were unsuccessful, and we did not obtain mutant iPSC clones. Following this negative outcome, we hypothesized that *ZNF827* is probably an essential regulator involved in cell survival of pluripotent cells. In support of this, we found *ZNF827* to be highly constrained against loss-of-function mutations (pLI = 1, gnomAD database v4) [49] and, therefore, be under a strong selection pressure with very few deleterious mutations found in large sequencing datasets. Similarly, overexpression of *ZNF827* resulted in major cell death after a few days, limiting further explorations of its function. Finally, our work leveraged genetic data and *in vitro* cell models, which limits the extrapolation of our findings to more complex cellular and physiological systems to fully address the role this gene might play in vascular remodeling and cardiovascular disease onset. Although we observed an effect of *ZNF827* on cellular proliferation and migration in SMCs, we did not assay in detail the effect on more complex pathways such as autophagy and insulin signaling.

In summary, our work provides a first exploration of the regulatory mechanisms at *ZNF827*, a pleiotropic genetic risk locus involved in diverse forms of ischemic heart disease, blood pressure, and aorta dimensions traits. Our results suggest several plausible molecular avenues involving major regulatory mechanisms with high relevance to vascular remodeling.

## Clinical perspectives

- Genome-wide association studies revealed ZNF827 as a genetic risk locus for SCAD, in addition to CAD, blood pressure, and aortic dimension. However, the molecular mechanisms underlying these genetic associations remain unexplored.
- We found that the causal SCAD risk allele at ZNF827 genetic risk locus created a binding site for the NF1 family transcription factor and was associated with increased expression of ZNF827. Potentially, it is affecting the expression of downstream genes involved in vascular pathologies such as TNFRS11B and CKAP4 in vascular SMCs and fibroblasts.
- ZNF827 transcriptional function suggests its role as a regulatory hub in SMC phenotypic modulation involved in arterial dissection. This molecular mechanism is a promising target for further exploration in more complex models of cardiovascular disease pathophysiology.

## Supplementary Material

Supplementary Figures S1-S13

Supplementary Tables S1-S4

## Data Availability

All supporting data are included within the main article and its supplementary files.

## Open Access

Open access for this article was enabled by the participation of INSERM in an all-inclusive Read & Publish agreement with Portland Press and the Biochemical Society.

## Abbreviations

AAdia: ascending aorta diameter
AAmax: ascending aorta maximum area
Aamin: ascending aorta minimum area
CAD: coronary artery disease
CKAP4: cytoskeleton-associated protein 4
GWAS: genome-wide association studies
LD: linkage disequilibrium
NF-1: nuclear factor 1 family
PP: pulse pressure
SMC: smooth muscle cell
SCAD: spontaneous coronary artery dissection
SBP: systolic blood pressure
TFBS: transcription factor binding site
ZNF827: zing finger protein 827
