# Supplementary material for: ZNF827 pleiotropic cardiovascular risk locus involves regulation by nuclear factor-1
Source: Clin Sci (Lond). 2026 May 5;140(5):791–804. doi: 10.1042/CS20257956 (PMC13161183; doi:10.1042/CS20257956)

Supplemental material

ZNF827 pleiotropic cardiovascular risk locus involves regulation by Nuclear factor-1

Yingwei Liu[1], Lu Liu[1,2], Asraa Esmael[1], Charlie London[1], Margaux-Alison Fustier[1], Adrien Georges*[1], Nabila Bouatia-Naji*[1]

[1]Université Paris Cité, Inserm, PARCC, F-75015 Paris, France
[2] Stanford Cardiovascular Institute, Palo Alto, CA, USA

Supplementary Figure Legends

Supplementary Figure 1

Local genomic plots represented the genetic association of common variants in *ZNF827* locus to pulse pressure (PP), ascending aorta maximal area (AAmax) and ascending aorta minimal area (AAmin). Top variant is indicated for each association, and color indicates the linkage disequilibrium (r2 in European population of 1000 Genomes reference panel) of all variants to top variant.

Supplementary Figure 2

A: UMAP plot of single-nucleus ATAC-seq. Clustering and annotation of single-nucleus was performed based on the pipeline and annotation strategy provided by authors[19]. B: Gene activity for marker genes associated to SMCs (*ACTA2, MYH11*) endothelial cells (*PECAM1*), fibroblasts (*LUM*), macrophages/monocytes (*CD14*) and T-cells (*RXRB*)

Supplementary Figure 3

Representative UMAP plots showing single cell populations profiled in single nuclei RNA-Seq analysis of diseased human coronary arteries [32] visualized using PlaqView[50], and featureplots of *ZNF827, NFIA, NFIB, NFIC* and *NFIX.*

Supplementary Figure 4

Network representation of female-biased gene-regulatory networks in gene expression from human carotid arteries [40]. Key networks drivers are highlighted in pink for MAGENTA subnetwork (**A**) and in black for BLACK subnetwork (**B**). Genes encoding transcription factors of NF-1 are highlighted by red circles, with NFIB as a key driver of MAGENTA subnetwork and *NFIA* as a key driver of BLACK subnetwork, while NFIX was also part of BLACK subnetwork. Figure adapted from Diez-Benavente et al.[40]

Supplementary Figure 5

Bar plot representation of transcription factor motif enrichment (*P*-value of hypergeometric enrichment - HOMER) in SMC-specific open chromatin region in atherosclerotic human coronary arteries [19]. Top 20 enriched motifs are represented. Bar color indicates the corresponding transcription factor family for each motif. Canonical transcription factor motif logo for each family was obtained from HOCOMOCO v13 database.

Supplementary Figure 6

Normalized expression of pluripotent stem cell markers coding genes (*NANOG*, *SOX2*, and *POU5F1*), mesenchymal stem cell markers coding genes (*SNAI1*, and *PDGFRB*), vascular smooth muscle cell markers coding genes (*ACTA2*, *TAGLN*, *MYH11*, and *SMTN*) and *ZNF827* gene in iPSCs and along differentiation to SMCs in iPSCs (day 0) and along differentiation to SMCs (days 3 to 24).

Supplementary Figure 7

Relative expression of *ZNF827* mRNA and mRNAs for NF-1 factors in iPSCs-SMCs transfected for 48h with non-target control siRNA or siRNA targeting *NFIA*, *NFIB*, *NFIC*, *NFIX* or all 4 NF-1 factors. P-values for unadjusted Wilcoxon rank sum test are indicated.

Supplementary Figure 8

Relative expression of *ZNF827* in iPSCs-SMCs and fibroblasts under siRNA non-target control or *ZNF827* knockdown condition.

Supplementary Figure 9.

Expression of SMC markers following *ZNF827* knockdown. Classical SMC markers, smooth muscle α-Actin (α-SMA), Transgelin, smooth muscle myosin heavy chain (SM MHC) and Calponin were assayed using Western Blot 48h post-transfection in iPSC-derived SMCs with negative control (Ct) siRNA or siRNA targeting *ZNF827*. Position of molecular weight markers are indicated on the left.

Supplementary Figure 10

A-B: Volcano plot representation of differential expression following *ZNF827* knockdown in iPSC-SMCs (**A**, 2 clones, 3 replicates each) and BJ Fibroblasts (**B**, 3 replicates). Log2 Fold Change is represented on *x* axis, while P-value is represented on *y* axis (log scale). Differentially expressed genes (*P.adj* < 0.05) are highlighted in blue (downregulated genes) or red (upregulated genes). **C:** Venn diagram illustrating the proportion of differentially expressed genes shared between SMCs and fibroblasts or restricted to one cell type

Supplementary Figure 11

Barplot representation of gene ontology pathways enriched amongst genes downregulated or upregulated following *ZNF827* knockdown in BJ fibroblasts. Top 20 pathways are represented. Bar color represent the adjusted P-value.

Supplementary Figure 12

A. Measurement of cell viability (mean±SEM,) of BJ fibroblasts treated with control siRNA (grey dots, 16 replicates) and *ZNF827* targetting siRNA (red triangles, 32 replicates The p-value for unadjusted Wilcoxon rank sum test is indicated at each time point. **B.** Measurement of cell viability (mean±SEM,) of iPSC-derived SMCs treated with control siRNAs (grey dots, 18 replicates) and *ZNF827* targetting siRNAs (red triangles, 18 replicates). The p-value for unadjusted Wilcoxon rank sum test is indicated at each time point.

Supplementary Figure 13:

Measurement of cell migration using Transwell assay. iPSC-derived SMCs were transfected with siRNAs targeting ZNF827 or control siRNA for 24h. 10000 cells were seeded in each well of a Transwell plate (8µM pore size) in presence or absence of 0.2µg/mL PDGF-BB added to culture medium. Migrating cells were stained with Crystal Violet and counted using a visible light microscope. 4 fields from 6 independent wells were counted. The p-values for unadjusted Wilcoxon rank sum tests are indicated.

Supplementary Tables Legends

Supplementary Table 1

SNP proxies at *ZNF827* locus. All SNPs in high linkage disequilibrium ($r^2>0.7$) of SCAD lead SNP (rs1507928) in the European population of 1000Genomes reference panel are indicated. Proxies were retrieved using LDlink webserver.

Supplementary Table 2

Prediction for rs13128814-dependent transcription factor binding sites. Differential affinity of rs13128814 alleles with transcription factor binding sites from HOCOMOCO database (v12) were estimated using PERFECTOS-APE package. Approximate P-values for both variants were estimated using threshold method [49].

Supplementary Table 3

Differential gene expression after *ZNF827* knockdown. Mean expression, Log2 Fold Change, P-value and adjusted P-value were estimated using DESeq2 package, taking into account all samples, samples from BJ fibroblasts and samples from iPSC-derived SMCs. All genes with adjusted P-value below 0.05 taking into account all samples are indicated.

Supplementary Table 4

Gene ontology enrichment in *ZNF827* target genes. Enrichment of gene ontology terms for 429 consistent ZNF827 target genes was estimated using clusterprofiler R package.

Supplementary Figure 1

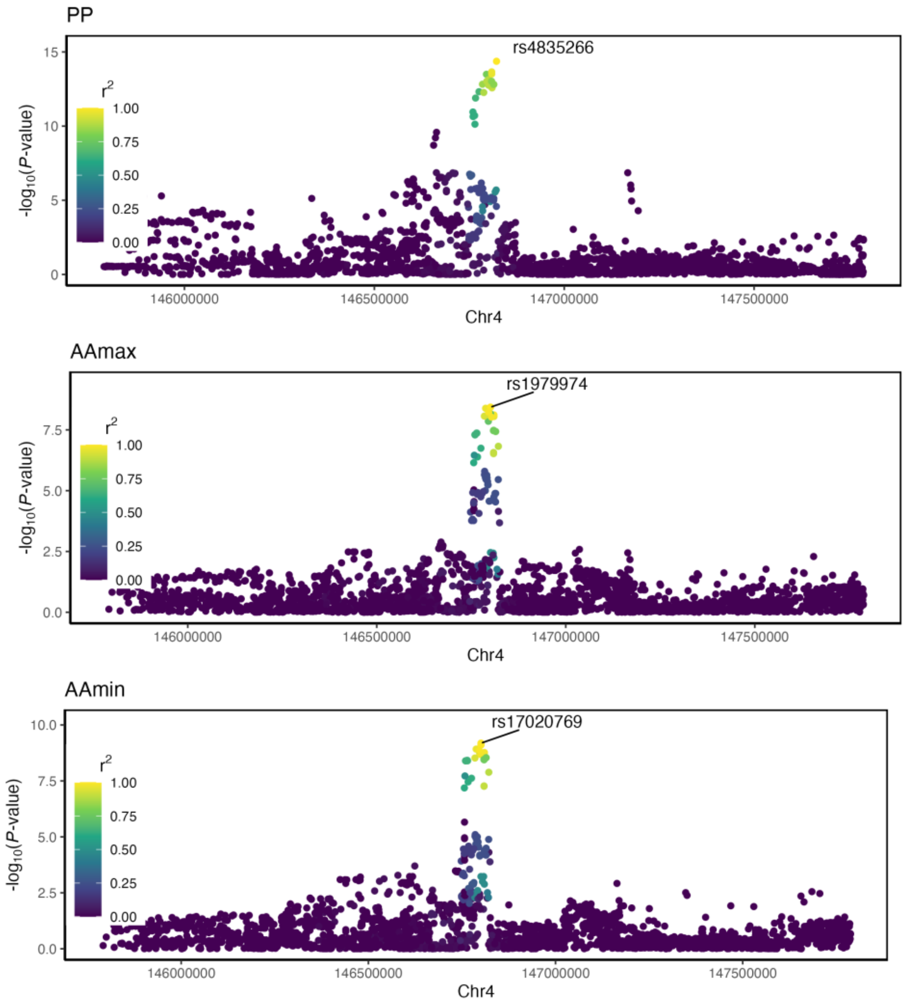

Supplementary Figure 2

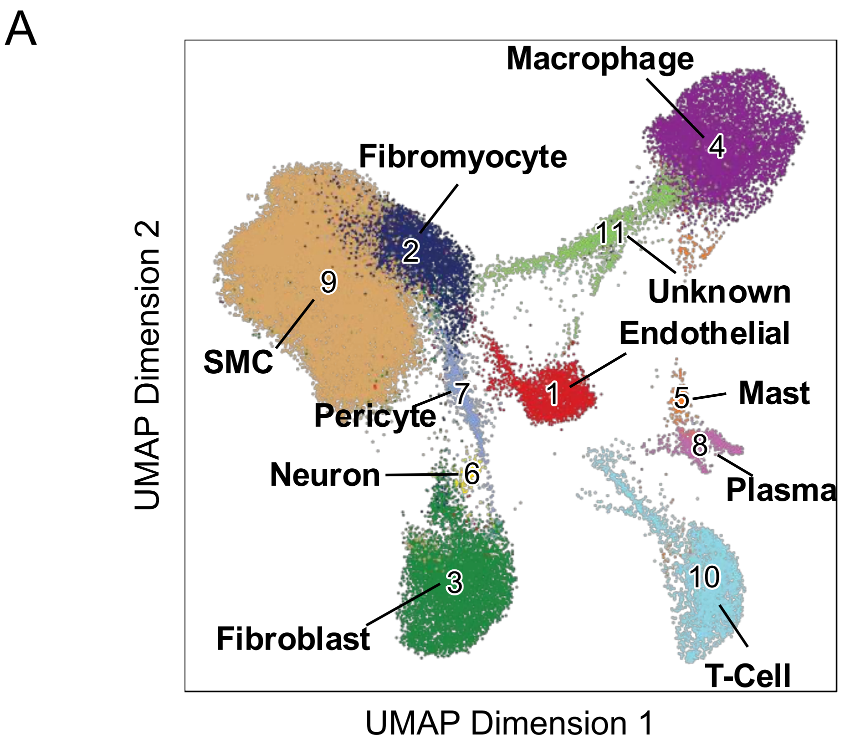

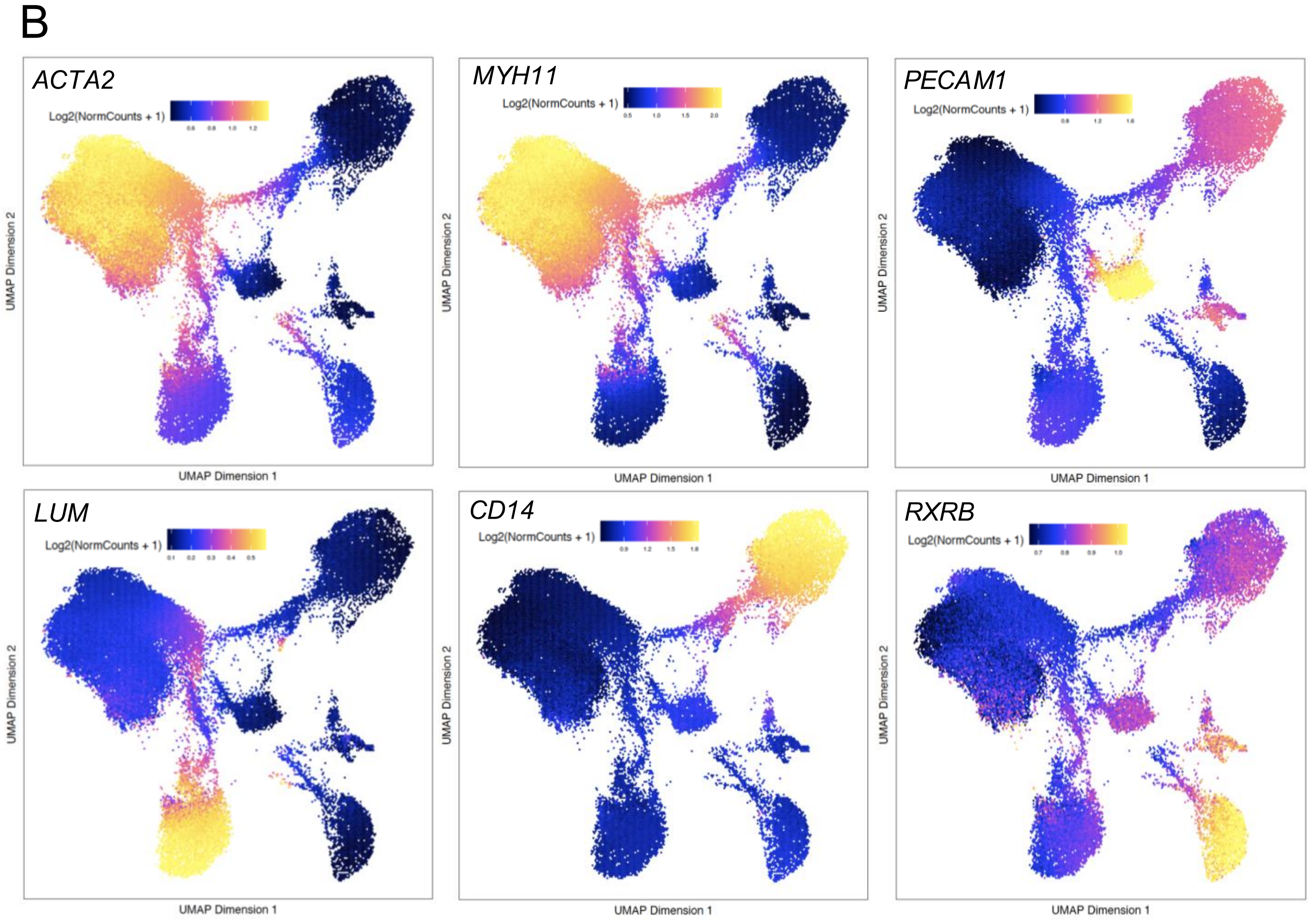

Supplementary Figure 3

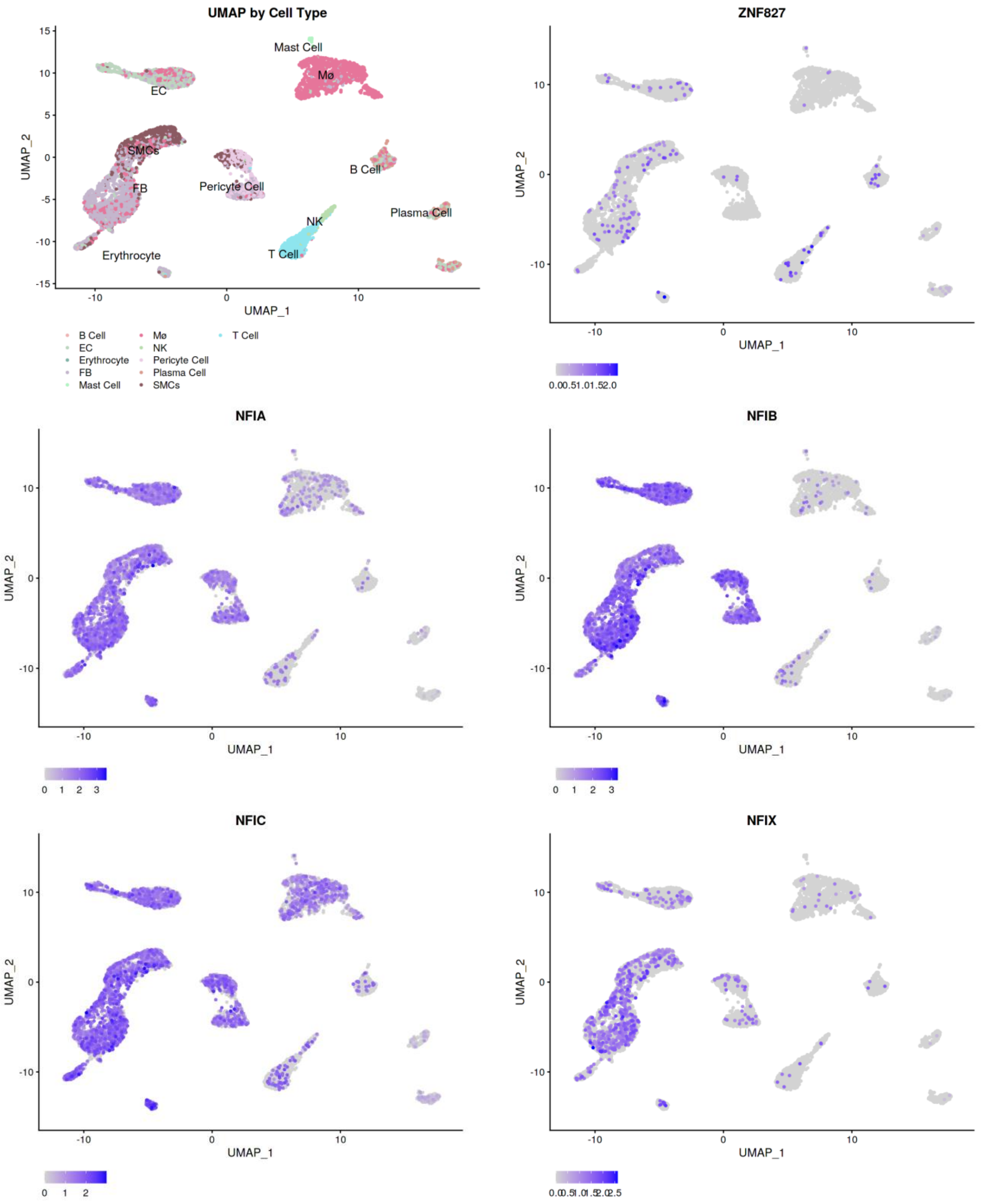

Supplementary Figure 4

A

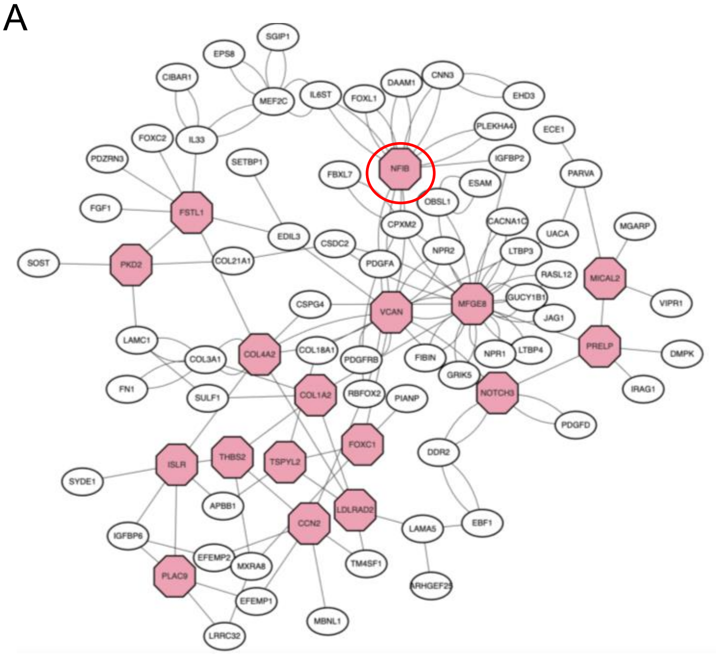

B

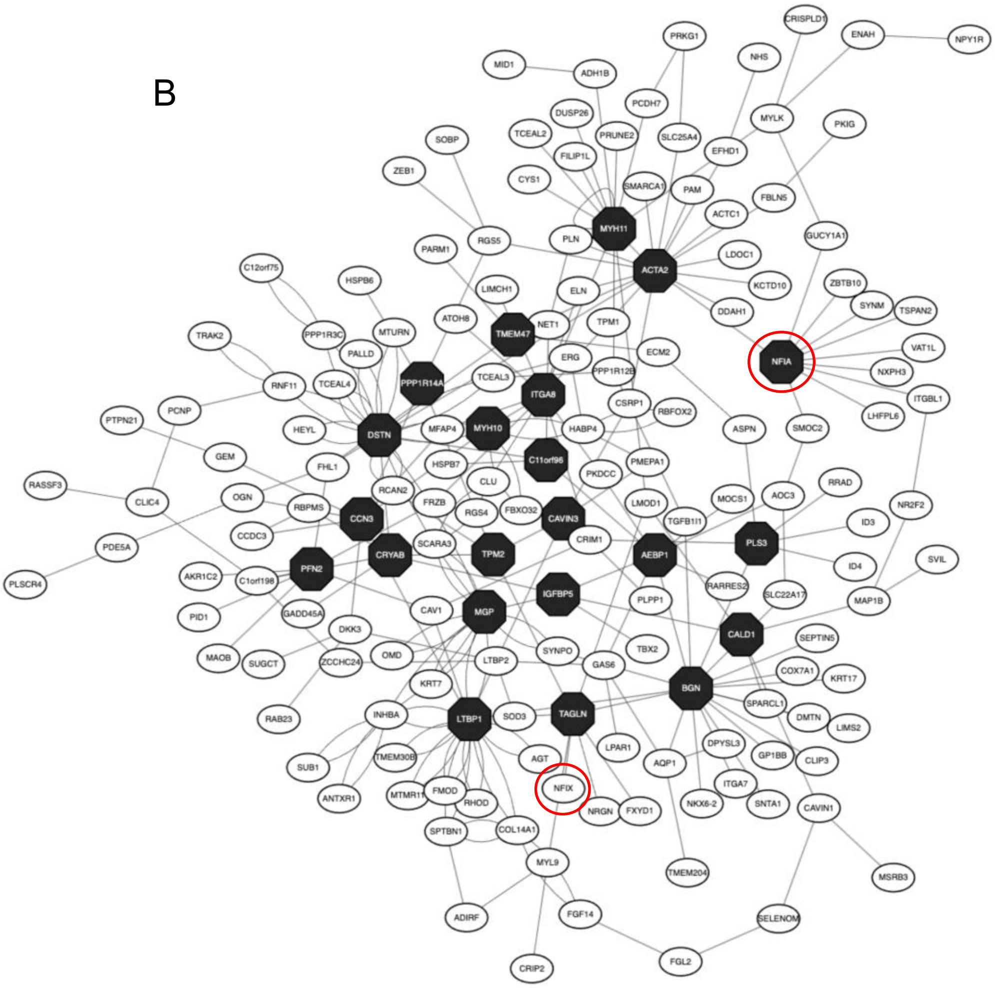

Supplementary Figure 5

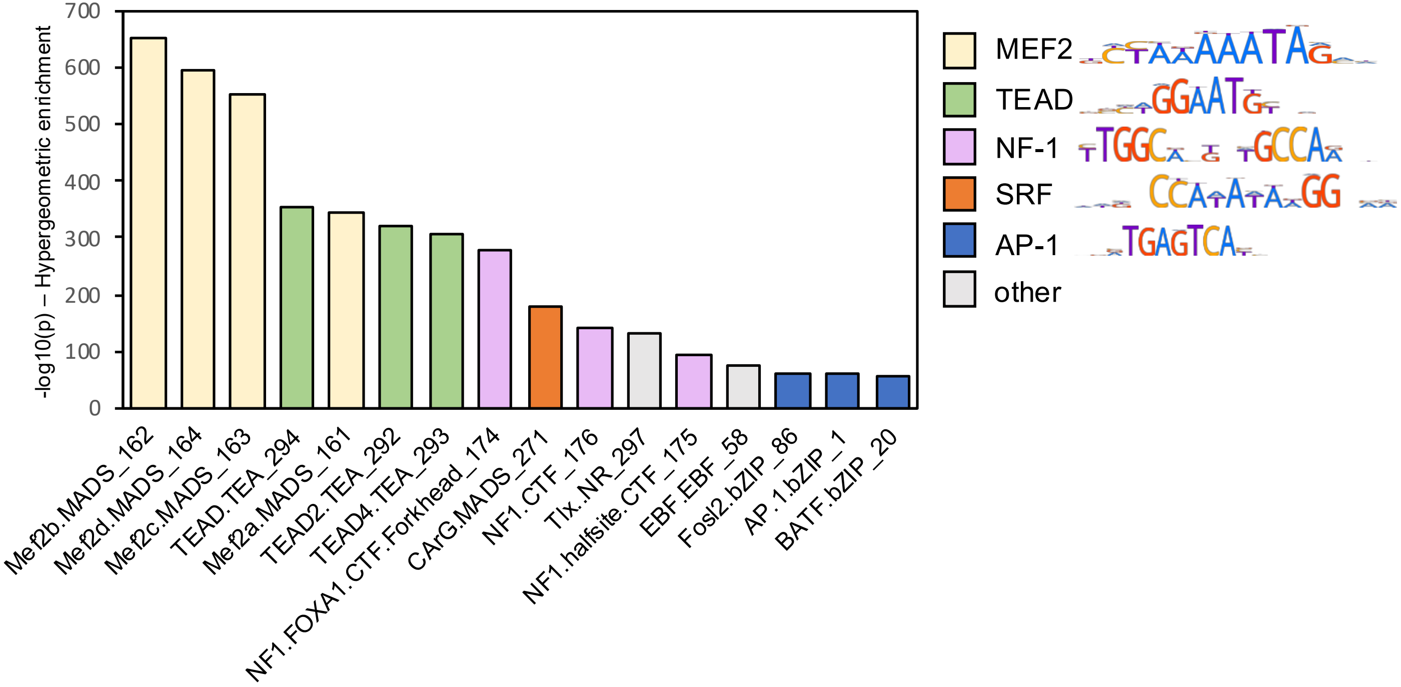

Supplementary Figure 6

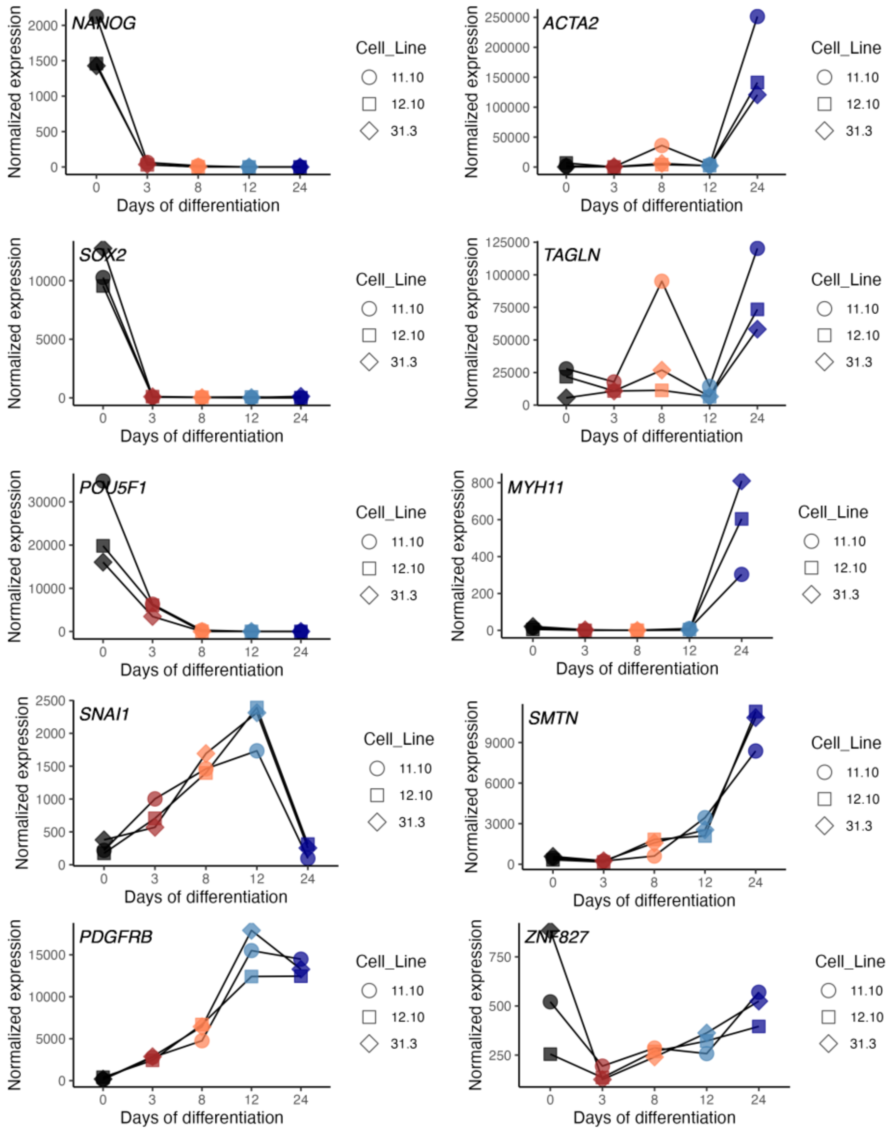

Supplementary Figure 7

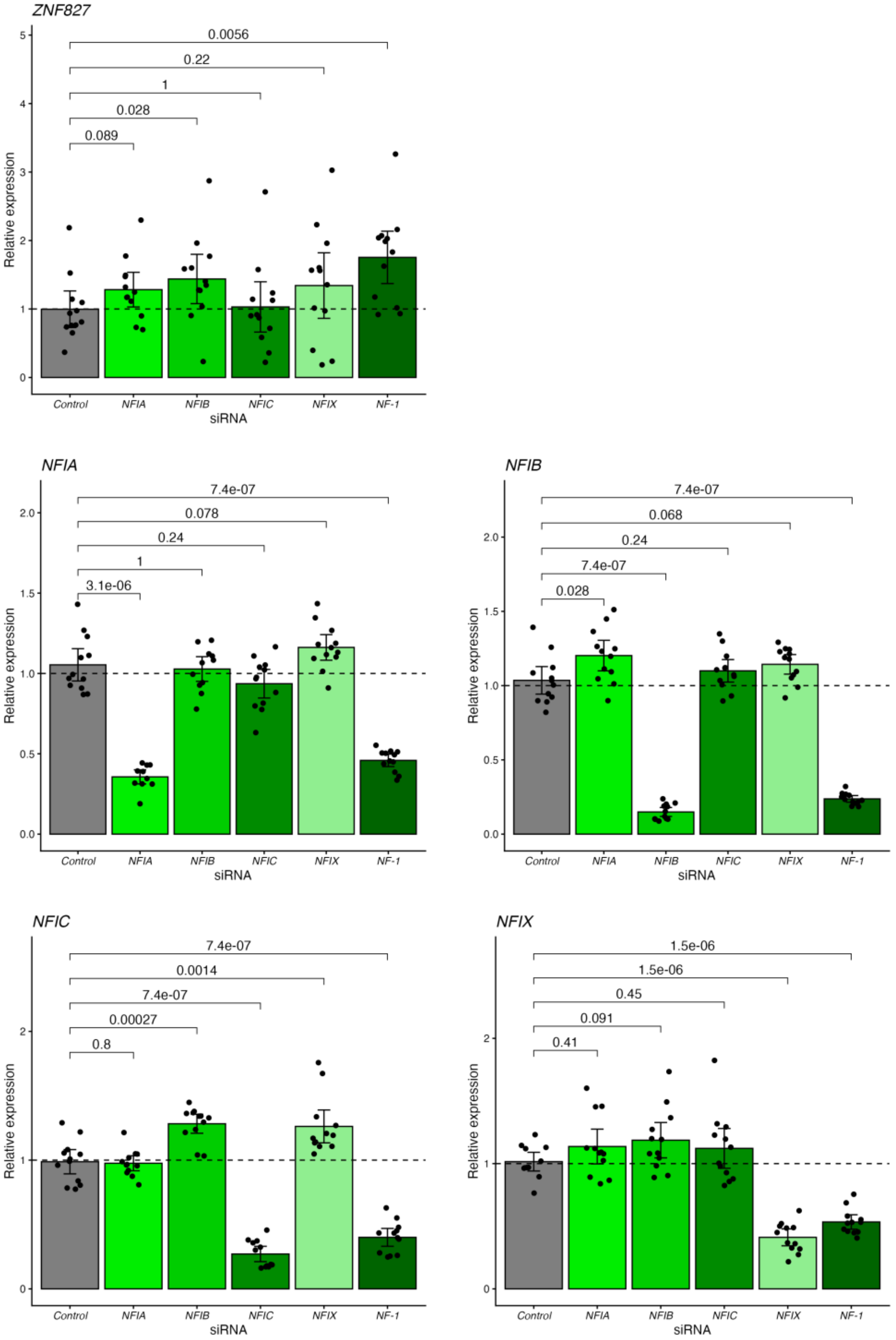

Supplementary Figure 8

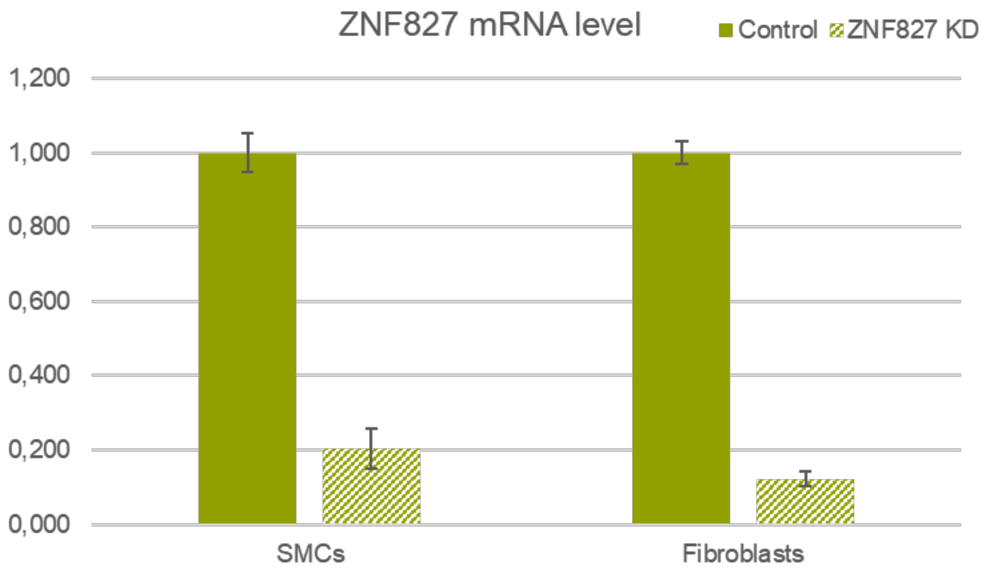

Supplementary Figure 9

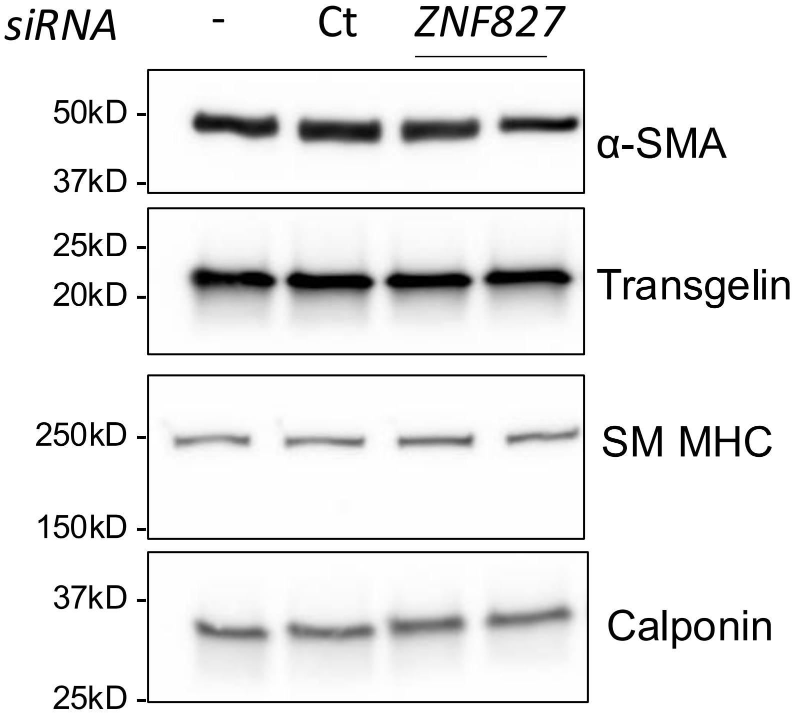

Supplementary Figure 10

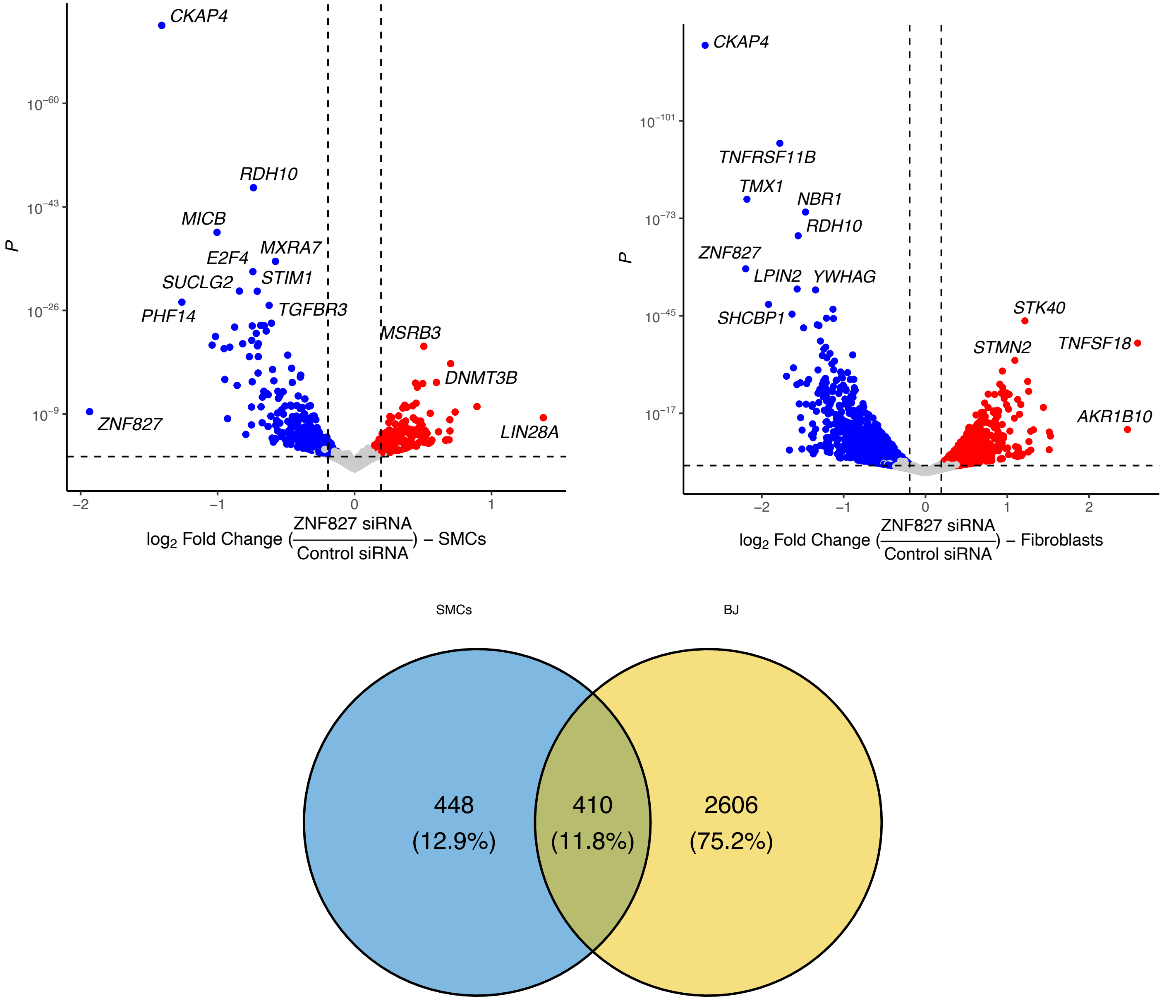

Supplementary Figure 11

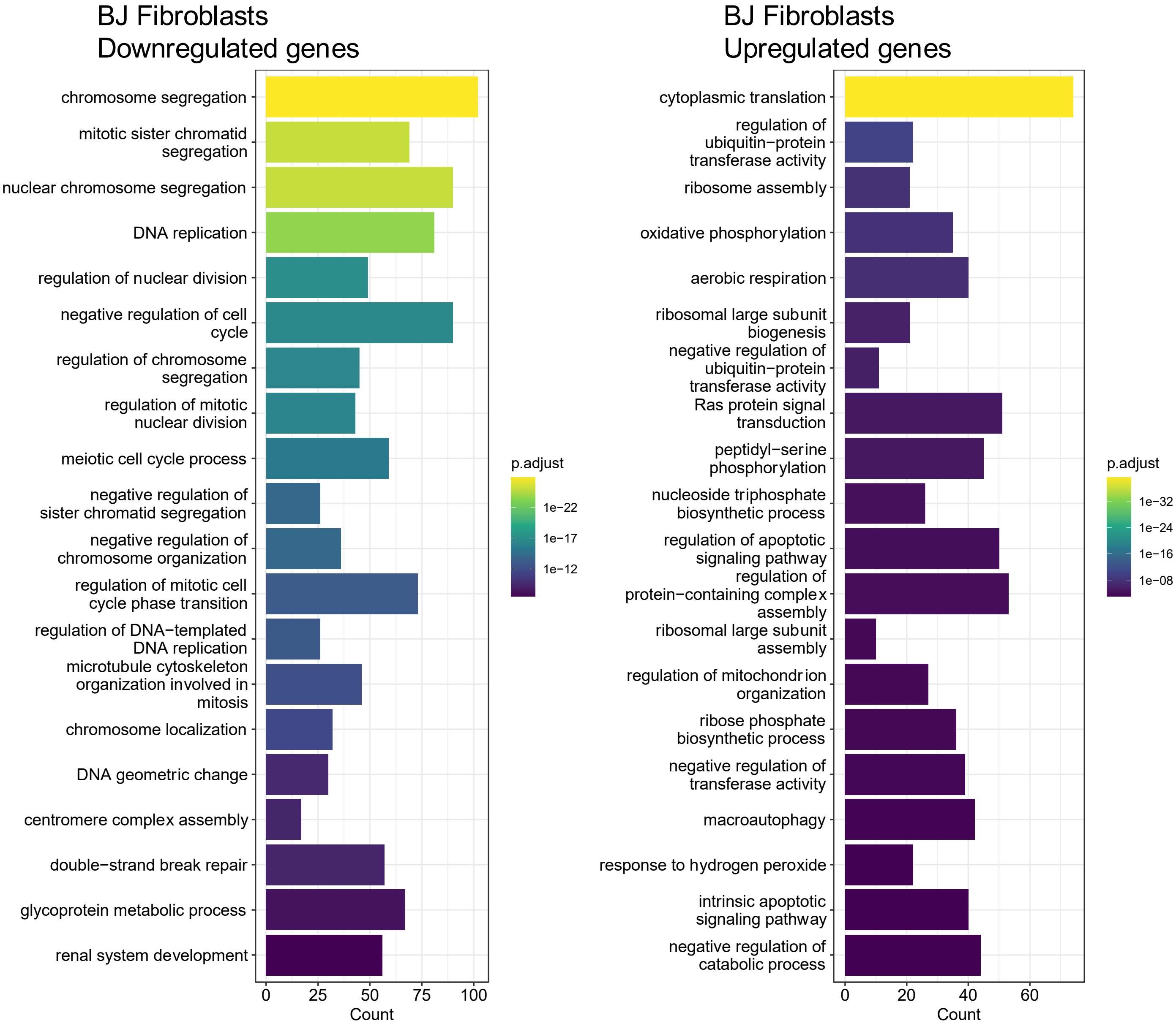

Supplementary Figure 12

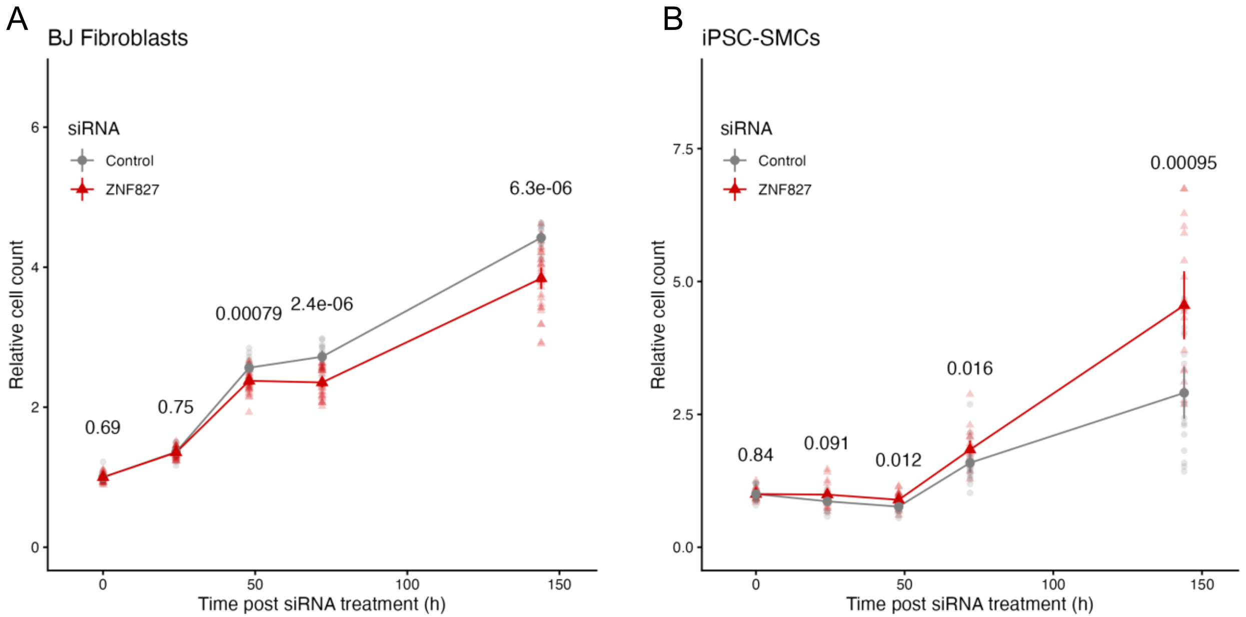

Supplementary Figure 13

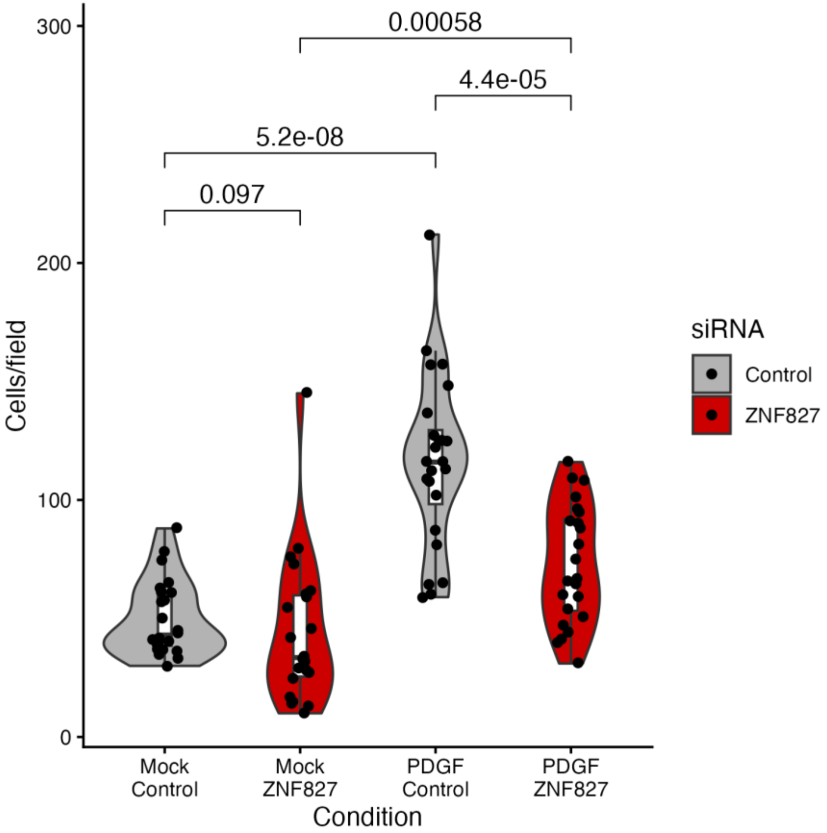

Supplement: Supplementary Figures S1-S13 [file CS-2025-7956_supp.pdf]
